# Ring-opening polymerization of emulsion-templated deep eutectic system monomer for macroporous polyesters with controlled degradability†

**DOI:** 10.1039/d3lp00232b

**Published:** 2024-02-05

**Authors:** Martín Castillo-Santillan, Priscila Quiñonez-Angulo, Dina Maniar, José Román Torres-Lubian, María C. Gutiérrez, Théophile Pelras, Albert J. J. Woortman, Qi Chen, María Guadalupe Pérez-García, Katja Loos, Josué D. Mota-Morales

**Affiliations:** a Centro de Física Aplicada y Tecnología Avanzada, Universidad Nacional Autónoma de México Querétaro QRO 76230 Mexico jmota@fata.unam.mx; b Macromolecular Chemistry and New Polymeric Materials, Zernike Institute for Advanced Materials, University of Groningen 9747AG Groningen The Netherlands; c Centro de Investigación en Química Aplicada (CIQA) Saltillo Coahuila 25294 Mexico; d Instituto de Ciencia de Materiales de Madrid (ICMM), Consejo Superior de Investigaciones Científicas (CSIC) Cantoblanco 28049 Madrid Spain; e Universidad de Guadalajara, Centro Universitario de Tonalá Tonalá Jalisco 45425 Mexico

## Abstract

Biodegradable polyesters with interconnected macroporosity, such as poly(l-lactide) (PLLA) and poly(ε-caprolactone) (PCL), have gained significant importance in the fields of tissue engineering and separation. This study introduces functional macroinitiators, specifically polycaprolactone triol (PCL_T_) and polyethylene glycol (PEG), both OH-terminated, in the solventless ring-opening polymerization (ROP) of a liquid deep eutectic system monomer (DESm) composed of LLA and CL at a 30 : 70 molar ratio, respectively. The macroinitiators selectively initiate the organocatalyzed ROP of LLA in the DESm during the first polymerization stage, thereby modifying the PLLA architecture. This results in the formation of either branched or linear PLLA copolymers depending on the macroinitiator, PCL_T_ and PEG, respectively. In the second stage, the ROP of the CL, which is a counterpart of the DESm, produces PCL that blends with the previously formed PLLA. The insights gained into the PLLA architectures during the first stage of the DESm ROP, along with the overall molecular weight and hydrophobicity of the resulting PLLA/PCL blend in bulk, were advantageously used to design polymerizable high internal phase emulsions (HIPEs) oil-in-DESm. By incorporating a liquid mixture of DESm and macroinitiators (PCL_T_ or PEG), stable HIPE formulations were achieved. These emulsions sustained the efficient organocatalyzed ROP of the continuous phase at 37 °C with high conversions. The resulting polymer replicas of the HIPEs, characterized by macroporous and interconnected structures, were subjected to a degradation assay in PBS at pH 7.4 and 37 °C and remained mechanically stable for at least 30 days. Notably, they exhibited the capability to sorb crude oil in a proof-of-concept test, with a rate of 2 g g^−1^. The macroporous and interconnected features of the polyHIPEs, combined with their inherent degradation properties, position them as promising degradable polymeric sorbents for efficient separation of hydrophobic fluids from water.

## Abstract 2

Multifunctional macroinitiators in the ROP of a DESm allow fine-tuning the properties of the resulting polyesters. This capability enables control over the degradability, M_n_, and polymer architectures of macroporous polyesters for oil sorption.

## Graphical abstract

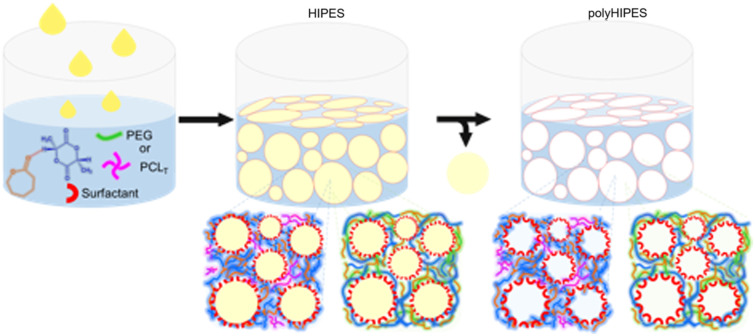

## Introduction

Macroporous polymers have found widespread use in various advanced applications, such as biomaterials,^1^ tissue engineering scaffolds,^2,3^ separation membranes,^4^ energy storage,^5^ and substrates facilitating specific surface-related phenomena.^6,7^ These applications stem from the enlarged surface area and the three-dimensional arrangement of interconnected pores, which enable efficient mass transport and specialized interfaces for reactions and interactions with target molecules.

One method to achieve such three-dimensional and interconnected macroporosity is through emulsion templating using high internal phase emulsions (HIPEs).^8^ HIPEs are biphasic systems in which the internal phase volume fraction exceeds 74%, facilitating the formation of densely packed micrometer-sized polyhedral droplets. These droplets stabilized by surfactants act as a template and are separated by a thin layer of continuous phase containing monomers.^6,9,10^ The utilization of a broad range of polymerization reactions, which leads to greater diversity in macromolecular structures, helps solidify the continuous phase of HIPE. Subsequent removal of the internal phase results in well-defined macroporous polymers with interconnected three-dimensional morphologies, known as polyHIPEs.

Macroporous polyesters stand out among other porous polymers because of their good biocompatibility and inherent degradability. These qualities make them the preferred polymer for applications such as biomaterials and other scenarios requiring degradability, whether as an integral aspect of performance or at the end of the lifecycle to mitigate pollution. Polyesters, such as polycaprolactone (PCL) and poly(l-lactide) (PLLA), are among the most important and widely used polymers due to their cleavable bonds through hydrolysis.^11,12^ However, the synthesis of these macroporous polyesters through the ring-opening polymerization (ROP) of monomer precursors in the continuous phase of HIPEs has been challenging.

Examples of ROP of monomers forming the internal phase of HIPEs to produce macroporous interconnected polyesters are scant. The research group of Srivastava was the first to report a single-step process for producing a porous polyester *via* enzyme-mediated ROP of a HIPE.^13^ On the other hand, ε-caprolactone (CL) has also been employed as a monomer precursor in the continuous phase of polymerizable nonaqueous HIPEs to produce macroporous crosslinked PCL at high temperatures and employing metal-based catalysts.^2,3,10,14,15^ Recurrent issues in polymerizing HIPEs to prepare macroporous polyesters include the moisture-sensitive polymerization mechanisms and the high temperatures typically employed in ROP, which are detrimental to HIPE stability and its template role.

One successful approach for polyHIPE preparation employing the ROP mechanism was the introduction of nonaqueous HIPEs, where deep eutectic system monomers (DESm) composed of l-lactide and CL constituted the polymerizable continuous phase. In this approach, LLA-CL DESm, at a molar ratio of 30 : 70, respectively, with a melting temperature (*T*_m_) of −19 °C, played the role of the continuous phase in a nonaqueous HIPE. The emulsion was polymerized to produce PLLA/PCL macroporous polyester blends.^16^ The liquid nature of the DESm was advantageous, facilitating the creation of highly stable HIPEs under ambient conditions.^17^ Therefore, in the pursuit of well-defined macroporous polymers with interconnected three-dimensional morphologies, the challenge has been to control the properties of the polyesters to achieve polyHIPEs with adjustable biodegradability, morphology, and mechanical properties.

In this study, we investigated the incorporation of macroinitiators in the ROP of a DESm composed of l-lactide and ε-caprolactone to improve the properties of the resulting polyesters, which can be translated into the preparation of polyHIPEs. The macroinitiators selectively initiate the organocatalyzed ROP of LLA in the first polymerization stage, yielding branched or linear PLLA copolymers depending on the macroinitiator, PCL_T_ and PEG, respectively. Blending PCL produced in the second stage of ROP with branched PCL_T_-*b*-PLLA or linear PEG-*b*-PLLA copolymers allows for the fine-tuning of hydrophobicity and thermal properties, both in bulk and in polyHIPE, thereby improving the overall performance of PLLA/PCL polyHIPEs as crude oil sorbents. The resulting polyesters, which contain branched PCL_T_-*b*-PLLA or linear PEG-*b*-PLLA copolymers and PCL, exhibit structural stability in PBS solution at pH 7.4 and 37 °C for 30 days and effectively sorb crude oil without collapsing. Due to their high macroporosity, interconnected morphology, compostability, absence of metal-based catalysts, and low polymerization temperature during their preparation, polyester-based polyHIPEs represent a promising class of biodegradable macroporous materials for separation processes.^16^ To demonstrate the overall performance of PLLA/PCL polyHIPEs as crude oil sorbents, a proof-of-concept experiment showing the potential application was performed with competitive figures.

## Experimental section

### Materials

l-Lactide (LLA, 98%), 1,8-diazabicyclo [5.4.0] undec-7-ene (DBU, 98%), polyethylene glycol (PEG, *M*_n_ = 6000 g mol^−1^), methanesulfonic acid (MSA, 99.5%), polycaprolactone triol (PCL_T_, *M*_n_ ≈ 900 g mol^−1^) and Pluronic® F-127 [poly(ethylene oxide)-*b*-poly(propylene oxide)-*b*-poly(ethylene oxide) triblock copolymer, *M*_n_ ≈ 12 600 g mol^−1^] were obtained from Sigma-Aldrich. ε-Caprolactone (CL, 97%) was obtained from Thermo Fisher Scientific. Tetradecane (>99.0%) was acquired from TCI Europe N.V. Absolute ethanol (EtOH, AR), methanol (MeOH, AR), and chloroform (CHCl_3_, >98%) were purchased from Biosolve Chemicals. *n*-Hexane (>99.0%) was obtained from Macron Fine Chemicals. Sweet crude oil (<0.5% sulfur) of the Rotterdam field was provided by Nederlandse Aardolie Maatschappij (NAM). All materials were used without further purification.

### Methods

#### Deep eutectic system monomer (DESm) synthesis

The deep eutectic system monomer (DESm) was prepared by mixing LLA with CL at a 30 : 70 molar ratio at 90 °C to obtain a clear and homogeneous liquid, following the methodology reported.^16,18^ To refer to DESm composed of 30 : 70 molar ratio LLA-CL, respectively, the nomenclature LLA_30_-CL_70_ will be used.

#### Synthesis of PLLA and PCL polymer blends

The synthesis of polyesters was carried out by the sequential and organocatalyzed ROP at 37 °C in bulk of LLA_30_-CL_70_ DESm. A solution of DBU (organocatalyst, [CAT] = 2.92 wt%) and BnOH (initiator, [In] = 2.1 wt%), with respect to DESm, in a proportion [CAT : In] = [1 : 1] mol%, was added to the DESm mixture with constant stirring. Subsequently, 3 wt% MSA (co-organocatalyst) was added 1 min after starting the reaction, following previous protocols for sequential ROP of DESm.^18,19^ The final polyesters were named as PLLA/PCL blend.

The previous method was used to obtain the PLLA homopolymer by stopping the reaction at one minute,^16^*i.e.*, after completion of the first ROP of LLA (following the corresponding kinetics). After that, the samples were dissolved in chloroform and immediately precipitated in cold methanol. In the next step, the sample was centrifuged at 4500 rpm for 10 min, and the precipitate was dried. Finally, the resulting product was labeled as PLLA, with an approximate molecular weight of 1800 g mol^−1^.

#### Synthesis of polyesters of PLLA and PCL with varying macroinitiators

The synthesis of polyesters was carried out by sequential ROP at 37 °C in bulk of LLA_30_-CL_70_ DESm using two macroinitiators: polycaprolactone triol (PCL_T_) and polyethylene glycol (PEG), both OH-terminated. The LLA_30_-CL_70_ DESm mixture was heated at 60 °C with constant stirring for 10 min, which contained the macroinitiator, and then the first organocatalyst (DBU) (2.92 wt% with respect to the DESm) was added. Subsequently, after 1 min (different times did not affect the PCL *M*_n_), MSA (second organocatalyst, 3 wt% with respect to the DESm) was added to start the ROP of PCL. The obtained polyesters were named as PCL_T_-*b*-PLLA/PCL and PEG-*b*-PLLA/PCL when using PCL_T_ and PEG as macroinitiators, respectively.

After 24 h of polymerization, the obtained polyesters were purified with an excess of ethanol 3 to 5 times for each sample to remove all residual monomers and oligomers. The solid polyesters were dried at room temperature (RT) for 24 h. The experimental conversion (*X*_exp_) was determined by gravimetry, that is, the ratio between the final mass of the polymer and the initial mass of the monomers.

Kinetics studies of PCL_T_-*b*-PLLA/PCL and PEG-*b*-PLLA/PCL were prepared in individual vials and were terminated at different times to analyze the time-dependent polymerization.

#### Preparation of high-internal-phase-emulsions (HIPEs)

The continuous phase of HIPEs was prepared by adding PCL_T_ or PEG at 1 wt% with respect to LLA_30_-CL_70_ DESm and heated to 60 °C for 10 min, then cooled to RT. Afterward, the surfactant Pluronic® F-127 was added at 10 wt% with respect to DESm. The HIPEs were prepared by the dropwise (*ca.* 0.5 mL min^−1^) addition of tetradecane (dispersed phase) accounting for 80 vol% with respect to the solution of a mixture DESm-initiator-Pluronic® F-127 (continuous phase, 20 vol%) in a 10 mL glass vial at 25 °C with constant agitation at 2500 rpm until a white homogeneous and viscous emulsion was obtained. The HIPEs were stable (no phase separation) for up to 24 h at RT.

#### Production of macroporous polyesters (polyHIPEs)

The monoliths were synthesized by the ROP of the HIPE continuous phase with DBU (2.92 wt% with respect to the DESm) as an organocatalyst. The emulsion was kept under constant stirring for one minute, and the second catalyst MSA (3 wt% with respect to the DESm) was added. HIPEs were homogenized by vortexing for 2 min. The ROP of the continuous phase was carried out for 24 h at 37 °C. After polymerization, polyHIPEs (PHIPEs) were purified with an excess of *n*-hexane for 12 h to remove the oil phase (tetradecane) and with ethanol for 3 days by orbital mixing to remove oligomers, residual monomers, and surfactant. Finally, monoliths were dried at RT until reaching a constant weight and subsequently dried at 40 °C for 2 h.

#### Production of self-assembled particles

To produce the self-assembled particles of PEG-*b*-PLLA/PCL, 30 mg of the polymer sample was added to 200 mL of THF, and then water (*ca.* 2 mL) was added dropwise to the clear solution while stirring until a steady change to white was observed. THF was removed by evaporation while the medium was stirred for 24 h. Finally, a milky solution was obtained. It is noteworthy that the same methodology was followed for the PCL_T_-*b*-PLLA/PCL sample; however, the polymer precipitated in water due to the absence of hydrophilic segments.

#### Degradation test

Porous polyesters were subjected to degradation profile assays. The samples were incubated at 37 °C in phosphate-buffered saline (PBS) pH = 7.4, and the degradation test was conducted for 14 and 30 days. Then, the polyesters were removed from the medium and rinsed with ethanol to remove any PBS residues. Finally, the samples were dried at RT. The pH of the medium was measured, and the remaining mass of the samples was calculated by gravimetry.

#### Oil adsorption test

The absorption capacities of monoliths (*Q*) were determined in crude oil at RT. The monoliths were immersed in excess of crude oil until the equilibrium mass of crude oil was taken up. *Q* was determined by the equation *Q* = (*W* − *W*_0_)/*W*_0_, where *W*_0_ and *W* are the weights of the monoliths before and after absorption, respectively, and was expressed in terms of the mass of crude oil per gram of dry monolith (g g^−1^).

#### Polyester characterizations

The final polyesters were analyzed by ^1^H and ^13^C NMR (600 MHz) spectroscopies and were recorded on a Bruker Avance NEO 600 at 64 scans. Samples were dissolved in deuterated chloroform (CDCl_3_) as an internal reference.

Attenuated total reflection Fourier transform infrared spectroscopy. (ATR-FTIR) spectra were recorded on a Bruker VERTEX 70 spectrometer equipped with a platinum-ATR diamond single reflection accessory. The measurement resolution was 1 cm^−1^, and spectra were collected in the range of 4000–400 cm^−1^.

Differential scanning calorimetry (DSC) measurements were carried out on a TA Instruments Q1000 DSC. The analysis method for polyesters was performed on dry samples and with heating–cooling cycles. The analysis consisted of an initial cooling from RT to −70 °C at a scan rate of 10 °C min^−1^, keeping it at that temperature for 5 min, then increasing the temperature to 220 °C, and finally cooling to RT at the same scan rate.

The thermal stability and degradation behavior of the different samples were studied using thermogravimetric analysis (TGA) on a TA Instruments Discovery TGA 5500 using an aluminum pan. Thermograms were recorded in the temperature range of 30 °C to 700 °C at a heating rate of 10 °C min^−1^ under a nitrogen atmosphere.

The X-ray diffraction patterns (XRD) of powdered samples were carried out in a Bruker D8 Advance diffractometer with Cu Kα radiation (*λ* = 0.1542 nm) in the angular range of 5–40° (2*θ*) at RT.

The molecular weight distributions (*Đ*) were determined by size exclusion chromatography (SEC) equipped with a triple detector: Viscotek Ralls detector, Viscotek Viscosimeter model H502 and Schambeck RI2912 refractive index detector. The separation was carried out by two PLgel 5 μm MIXED-C and 300 mm columns from Agilent Technologies at 35 °C in THF (99% extra pure) stabilized with BHT as the eluent at a flow rate of 1.0 mL min^−1^. The molecular weight calculations were performed based on the universal calibration curve generated by narrow polydispersity polystyrene standards (Agilent and Polymer Laboratories) with the *M*_w_ values ranging from 645 to 3 001 000 g mol^−1^. The data acquisition was processed by using Viscotek Omnisec software version 5.0.

DOSY (Diffusion Order Spectroscopy) data were acquired using the pulse program *ledbpgp2s* installed in topspin 3.6.2 software from Bruker with 95 gradient levels with a linear increase from 2 to 95% using a gradient of strengths up to 54 G cm^−1^ and 8 transients. The diffusion delay (*Δ*) was 150 ms, and the length of the square diffusion encoding gradient pulse (*δ*) was 0.6 ms. Laplace transformations for generating the diffusion dimensions were obtained with the Bruker Biospin Dynamics Center using a least-squares fitting routine with Monte Carlo error estimation analysis.

The contact angle was recorded by an OCA20 (Data physics). The measurements were performed on polymer films prepared by solvent casting. The obtained polyesters were dispersed in CHCl_3_ and poured onto a glass plate, allowing the solvent to evaporate.

The surface morphology of PHIPEs was examined by scanning electron microscopy (SEM, Hitachi S-4700). The macroporous structure of the monoliths was observed by scanning electron microscopy (SEM, Fei NovaNanoSEM 650) at an accelerating voltage of 5 kV. All samples were gold-coated. The diameters of the pores were calculated by ImageJ analysis software as the average of 100 image readings. These values were used to estimate the degree of openness of porous polyesters by the application of the equation proposed by Pulko and Krajnc.^20^

Dynamic light scattering (DLS) measurements were conducted on a Malvern Panalytical Zetasizer Ultra system equipped with a helium–neon laser (*λ* = 633 nm) and an avalanche photodiode detector. The solutions (c ∼ 1 g L^−1^ in Milli-Q water) were measured at 25 °C in back scattering mode after 120 s equilibration time and using 30 cumulative recordings. Samples were recorded in triplicates and the results were analyzed with ZS Xplorer software.

TEM imaging was performed on a Philips CM120 transmission electron microscope equipped with a Lanthanum hexaboride filament and operated at an accelerating voltage of 120 kV. Images were acquired using a Gatan slow-scan 4K CCD camera. Specimen were prepared by deposition of 5 μL of the nanoparticle dispersion (c ∼ 1 g L^−1^ in Milli-Q water) onto a glow-discharged (15 s at 50 mA and 300 V) 400-mesh copper grid with carbon support film and adsorption for 1 min before blotting. Negative staining was applied before the specimen was fully dried, 5 μL of 2 wt% uranyl acetate staining solution was deposited onto the grid, immediately blotted and a new 5 μL drop of staining solution was deposited and left to adsorb for 1 min before blotting. TEM images were analyzed using Image J software, using the software brightness and contrast correction tools to enhance the general quality of the snapshots.

AFM imaging was conducted in standard tapping mode in air, using a Bruker Dimension 3100 system equipped with VTESPA-300 tapping mode cantilevers from Bruker. Samples (c ∼ 1 g L^−1^ in Milli-Q water) were spin-coated (4 000 rpm for 60 s) onto a freshly-cleaved mica disc (Ø = 9.5 mm, muscovite mica grade V-1 from Proscitech) and measured on the following day. Images were processed with Bruker NanoScope software and the software-imbedded cross-section tool was utilized to determine the dimensions of the nanoparticles.

The PHIPE density (*δ*_B_) was estimated by measuring the volume of monoliths with regular shapes. The total pore volume (*V*_T_) was estimated as 1/*δ*_B_ − 1/*δ*_W_, where *δ*_W_ is the wall density that corresponded to the bulk polyesters, PCL_T_-*b*-PLLA/PCL and PEG-*b*-PLLA/PCL blend density, *ca.* 0.9253 and 1.032 g mL^−1^, respectively.

## Results and discussion

### Ring opening polymerization of LLA_30_-CL_70_ DESm

**Scheme 1:**
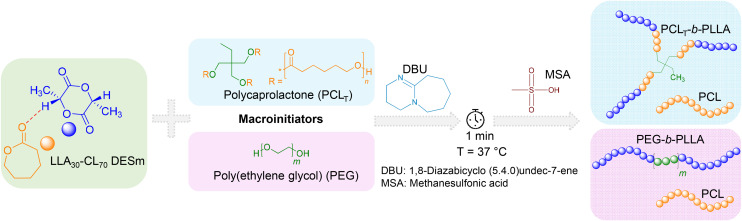
Sequential Ring-Opening Polymerization (ROP) of LLA_30_-CL_70_ DESm at 37 °C, varying the type of macroinitiator, PCL_T_ or PEG.

The sequential ROP of the LLA_30_-CL_70_ DESm at 37 °C in bulk yielded two polyester blends, PCL_T_-*b*-PLLA/PCL and PEG-*b*-PLLA/PCL, depending on the macroinitiator used (PCL_T_ or PEG, respectively). The macroinitiators accounted for 1 wt% with respect to DESm. The overall reaction is described in Scheme 1. In the first step, DBU organocatalyst (2.92 wt% with respect to the DESm) was added to the reaction mixture. DBU rapidly polymerized LLA in the DESm through the activation of both the alcohol and the monomer,^21^ where either PCL_T_ or PEG (OH-terminated) served as macroinitiators *via* the hydroxyl moieties. At this stage of the ROP, the average composition comprised PLLA blocks initiated by PCL_T_ or PEG, *i.e.*, branched PCL_T_-*b*-PLLA or linear PEG-*b*-PLLA dispersed in liquid CL, which was released from the liquid eutectic composition as the LLA ROP proceeded. It is noteworthy that PCL_T_ has *M*_n_ = 900 g mol^−1^, whereas PEG has *M*_n_ = 6000 g mol^−1^, and both generated liquid solutions in the DESm at the polymerization temperature; thus, the whole reaction mixture is liquid at RT, making all components readily available to polymerize. In the second step, MSA (second organocatalyst) was added to the reaction mixture, promoting the ROP of CL, where residual water played the role of initiator, as has been demonstrated in previous works.^16,18^ Thus, blends of PCL with branched PCL_T_-*b*-PLLA or linear PEG-*b*-PLLA were obtained depending upon the initiator used in the ROP, yielding polyesters with interesting properties, as will be discussed in the next sections.

**Fig. 1:**
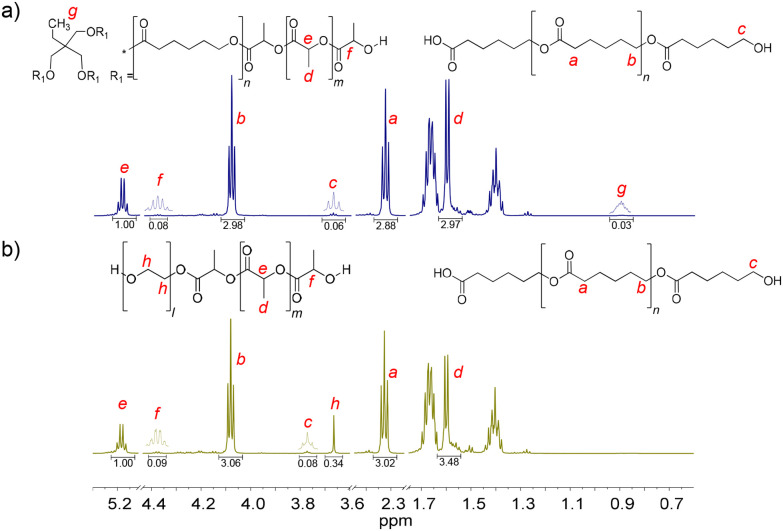
^1^H NMR spectra of the final products of the ROP of LLA_30_-CL_70_ DESm at 37 °C varying PCL_T_ or PEG as the macroinitiator of polyesters: (a) PCL_T_-*b*-PLLA/PCL, and (b) PEG-*b*-PLLA/PCL.

PCL_T_-*b*-PLLA/PCL and PEG-*b*-PLLA/PCL polyesters were first studied in bulk. The detailed procedure is described in the Experimental section. The presence of PLLA in PCL_T_-*b*-PLLA/PCL and PEG-*b*-PLLA/PCL was confirmed by ^1^H NMR, where the peak *H*_e_ = 5.17 is assigned to the methine (–OC**H**CH_3_–)_*n*_, and *H*_d_ = 1.66 ppm is assigned to the methyl (–CHC**H**_**3**_)_*n*_ repeating groups (see Fig. 1).^22^ Similarly, the presence of peaks *H*_b_ = 4.09 ppm and *H*_a_ = 2.33 ppm were identified as the typical repeating methylene groups of PCL.^16^ Additionally, the PLLA block in PCL_T_-*b*-PLLA/PCL and PEG-*b*-PLLA/PCL was confirmed by the terminal peaks *H*_f_ = 4.38 ppm assigned to the methine group (–C**H**(CH_3_)–OH).^16^ The presence of PCL in PCL_T_-*b*-PLLA/PCL was confirmed by the peak *H*_c_ = 3.67 ppm (Fig. 1a) and in PEG-*b*-PLLA/PCL with the peak *H*_c_ = 3.77 ppm (Fig. 1b), which are shifted to the low field due to the abundant presence of methylene groups corresponding to the repetitive units of ethylene glycol present in the polymer blend (Fig. S1 in the ESI†). The formation of PLLA and PCL in the polyesters was corroborated by ^13^C NMR spectroscopy. Fig. S2a in the ESI† shows the carbon peaks *C*_8_ = 169 and *C*_7_ = 64 ppm corresponding to the carbonyl (**C**OO–) and the repeated groups of methine (–**C**H(CH_3_))_*n*_ of PLLA, respectively, while the carbonyl (**C**OO–) of PCL *C*_1_ = 173 ppm was also identified.^16^

Furthermore, the presence of initiators, PCL_T_ and PEG, in PCL_T_-*b*-PLLA/PCL and PEG-*b*-PLLA/PCL polyesters was confirmed by ^1^H NMR. Specifically, PCL_T_ was identified by the signal *H*_g_ = 0.90 ppm attributed to the methyl terminal group (C**H**_**3**_CH_2_–) (see Fig. 1a).^23^ The *H*_h_ = 3.66 ppm signal was assigned to the methylene groups (–C**H**_**2**_C**H**_**2**_O–)_*n*_ of PEG.^24^ Fig. S2b in the ESI† shows the ^13^C NMR spectra of PEG-*b*-PLLA/PCL, where the carbon peaks at *C*_7_ = 64 ppm and *C*_2_ = 69 corresponding to repeated groups of methine (–C**H**(CH_3_))_*n*_ and methylene (–C**H**_**2**_O–)_*n*_, from PLLA and PCL, respectively, are observed.^16^ The presence of PEG in the polyesters was identified by the peak at 70 ppm, which corresponds to the methylene group (–C**H**_**2**_O–)_*n*_.^25^ Altogether, the ^1^H NMR and ^13^C NMR spectra of PCL_T_-*b*-PLLA/PCL and PEG-*b*-PLLA/PCL polyesters confirmed the role of PCL_T_ and PEG as macroinitiators in the ROP of LLA_30_-CL_70_ DESm.

Experimental conversions (*X*_exp_) were determined gravimetrically. The experimental conversion of PCL_T_-*b*-PLLA/PCL was 95% after 24 h, and for PEG-*b*-PLLA/PCL, it was 90% after 12 h.

Due to the presence of PLLA blocks and the PCL homopolymer in the polyester blends, *i.e.*, clear identification of the terminal *H*_f_ in PLLA and *H*_c_ in PCL (Fig. 1), it was possible to determine the molecular weight (*M*_n_) of PLLA blocks in branched and linear polyesters. Similarly, *M*_n_ was obtained for the PCL homopolymers resulting from the ROP of the CL in the DESm. The molecular weights of PLLA and PCL in the polyesters were determined by SEC and ^1^H NMR (Table 1) by eqn (S4)–(S6) in the ESI.†

**Table 1:** Chemical and thermal properties, and contact angle of the polyesters obtained by the ROP of LLA_30_-CL_70_ DESm at 37 °C in bulk, varying PCL_T_ or PEG as macroinitiator

Sample^a^	*F*_PLLA_ : *F*_PCL_^b^	*M*_n, PLLA_ c (g mol^−1^)	*M*_n, PCL_ c (g mol^−1^)	*M*_n_ d (g mol^−1^)	*Đ* d	*T*_m, PLLA_ e (°C)	*T*_m, PCL_ e (°C)	Contact angle (°)
PCL_T_-*b*-PLLA/PCL	51 : 49	1700	3840	6630	1.77	133	58.7	71
PEG-*b*-PLLA/PCL	40 : 60	1600	4370	6220	4.09	122	60.2	56

a The ROP LLA_30_-CL_70_ DESm was carried out using DBU and MSA as organocatalysts. The molar ratio of catalyst DBU and the initiator was 2.92 and 1 wt%, respectively, and the MSA was 3 wt%, all of them with respect to the DESm.

b The mole fraction of monomers (*F*_i_, where i = PLLA or PCL) was obtained by ^1^H NMR using eqn (S1)–(S3) in the ESI.†

c *M*_n_ was obtained by ^1^H NMR using eqn (S4)–(S6) in the ESI.†

d Combined *M*_n_ of PCL and PLLA obtained by SEC in THF.

e Thermal properties calculated by DSC.

The estimation of the mole fraction of monomers (*F*_i_, where i = PLLA or PCL) was obtained by ^1^H NMR in the PCL_T_-*b*-PLLA/PCL and PEG-*b*-PLLA/PCL samples. The data show differences in the composition between the expected molar ratio of LLA_30_-CL_70_ DESm and the final polyesters (Table 1) due to the loss of PCL oligomers during the purification process with ethanol, as has been observed in the ROP of DESm initiated with benzyl alcohol (BnOH).^19^

The ROP kinetics of LLA_30_-CL_70_ DESm varying the macroinitiator (PCL_T_ or PEG) were comparatively studied in section 2 in the ESI.† The methodology and formulations were similar to those previously reported, which were initiated by BnOH, pointing to the rapid polymerization of LLA during the first polymerization stage (1 min) followed by a steady polymerization of CL triggered by the MSA organocatalyst (Fig. S3 in the ESI†). This second stage follows first-order kinetics associated with the slope of the curve corresponding to the initiation rate constant (*k*^app^_p,CL1_) (Table S1 in the ESI†), showing that the polymerization presents the same behavior as the sequential ROP of LLA_30_-CL_70_ DESm initiated by BnOH. The relationship between the conversion of LLA_30_-CL_70_ DESm and the polymerization time follows the same increasing trend regardless of the macroinitiator used, reaching up to 90% at 24 h.

Size exclusion chromatography (SEC) was performed on the polyesters, and the traces are presented in Fig. S4 in the ESI.† The *M*_n_ values of the branched PCL_T_-*b*-PLLA/PCL and linear PEG-*b*-PLLA/PCL samples are summarized in Table 1. The combined *M*_n_ values of PCL and PLLA obtained by ^1^H NMR spectroscopy were slightly lower than those obtained by SEC (Fig. S10 in the ESI†). The differences arise from samples having different hydrodynamic hindrance volumes due to their different architectures and the additional interactions with the PCL homopolymers in the blends. In the case of the *M*_n_ values of the branched PCL_T_-*b*-PLLA/PCL (*M*_n_ = 6630 g mol^−1^), the PLLA branches initiated by PCL_T_ macroinitiator may increase the retention interactions in the SEC chromatography column. This *M*_n_ value surpasses the combined *M*_n_ of PCL_T_-*b*-PLLA and PCL as determined by ^1^H NMR (*M*_n_ = 5540 g mol^−1^).

However, in the case of PEG-*b*-PLLA/PCL with a linear architecture, these interactions are less pronounced, resulting in negligible differences between the weights obtained by ^1^H NMR and SEC. Thus, the final polyester blends eluted as broad distributions due to the compatibility between the polymers composing the blends; this behavior was also observed in the peak distributions of branched PCL_T_-*b*-PLLA/PCL and linear PEG-*b*-PLLA/PCL analyzed by ^1^H NMR diffusion-ordered spectroscopy (DOSY), as shown below.

**Fig. 2:**
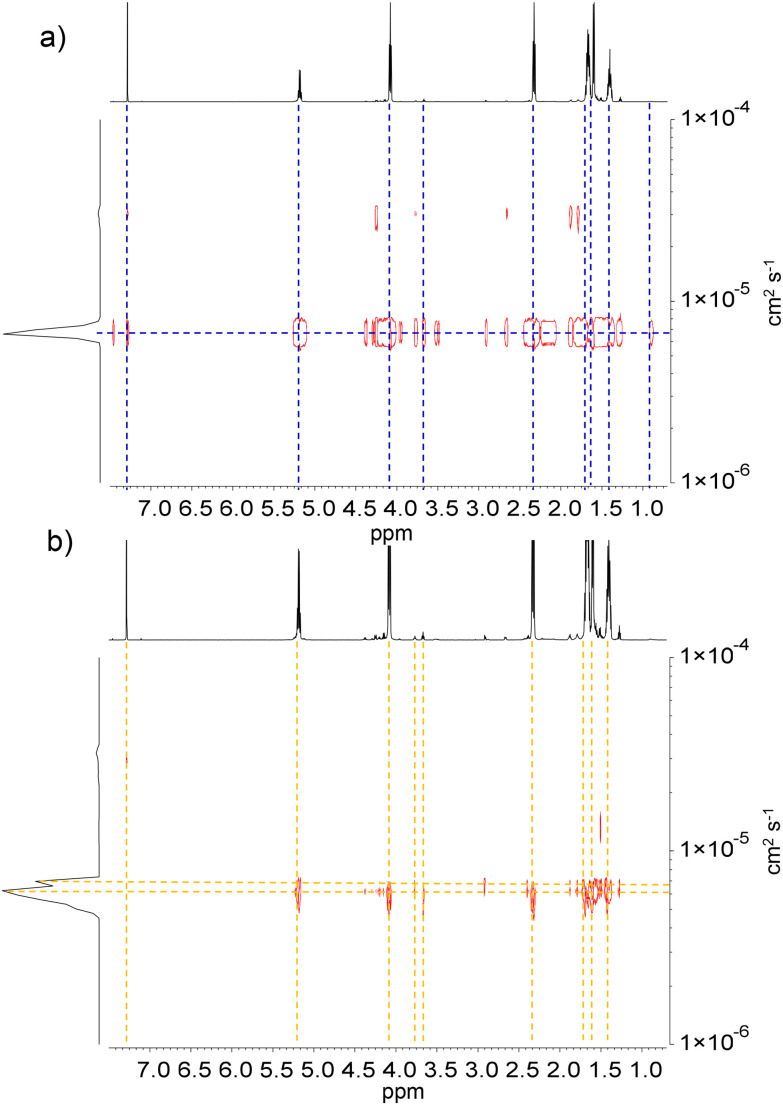
DOSY spectra of (a) PCL_T_-*b*-PLLA/PCL and (b) PEG-*b*-PLLA/PCL in CDCl_3_. The diffusion coefficient DM (cm^2^ s^−1^) is indicated for each component.

The PCL_T_-*b*-PLLA/PCL and PLLA-*b*-PEG/PCL polyesters were analyzed by DOSY spectroscopy. Fig. 2a shows the DOSY spectra of the polyester blends. The diffusion coefficient distributions of the polymer species were compared with the upper axis representing the corresponding ^1^H NMR spectra. A narrower diffusion coefficient was observed for PCL_T_-*b*-PLLA/PCL than for PEG-*b*-PLLA/PCL, which suggests a major influence of the polymer architecture due to the PEG macroinitiator hydrophilicity, *i.e.* more hydrophobic PCL_T_-*b*-PLLA/PCL diffuse as a single entity whereas PEG-*b*-PLLA/PCL behave as entangled polymers with broad and bimodal diffusion coefficients. The shoulder in the diffusion coefficient distribution of PEG-*b*-PLLA/PCL can be considered an effect of the PCL homopolymer being less compatible with the more hydrophilic PEG-*b*-PLLA/PCL block compared with the PCL_T_-*b*-PLLA/PCL, which has PCL in the macroinitiator. Although PEG has been reported to have better compatibility with PLLA than PCL,^26^ the incorporation of a PEG block could still act as a plasticizer^27^ that enhances the overall compatibility between the PLLA/PCL blends, as no polymer segregation occurred.

These findings point to PLLA block copolymers polymerized in the first ROP entangled with the PCL homopolymer from the second ROP. Ultimately, blending PCL with branched PCL_T_-*b*-PLLA or linear PEG-*b*-PLLA copolymers can be used to tune the hydrophobicity of the final materials.

The PEG-*b*-PLLA copolymer and PCL homopolymer had two different molecular weights, which resulted in different diffusion coefficients. This difference was confirmed by the broad *M*_n_ distribution obtained from SEC analysis, which has a dispersity index (*Đ*) of 4.09 (Table 1). As mentioned earlier, the PEG block in the copolymer played a critical role as a compatibilizer, affecting the interactions between PLLA and PCL.

The thermal properties of the obtained polyesters were studied by DSC, and the results are listed in Table 1. Fig. S5a in the ESI† shows the DSC thermograms during the first heating cycle of PCL_T_-*b*-PLLA/PCL and PEG-*b*-PLLA/PCL. The first peak at approximately 60 °C corresponds to the melting point (*T*_m_) of PCL (reported at approximately 62 °C),^28^ and the second peak corresponds to the *T*_m_ of PLLA (122–133 °C) embedded in PCL.^29^ Two endothermic peaks of PLLA in sample PCL_T_-*b*-PLLA/PCL were observed at 128 °C and 133 °C, suggesting the presence of PLLA chains of different *M*_n_ in a single PCL_T_ (trifunctional macroinitiator).^30^ Alternatively, it is possible that hydroxyl groups of PCL_T_ were not able to initiate the ROP of LLA, although hydroxyl groups in both PCL_T_-*b*-PLLA/PCL and PEG-*b*-PLLA/PCL by ATR-FTIR (Fig. S6 in the ESI†) are negligible, meaning that the remaining hydroxyl from PCL_T_ is not detected.

On the other hand, in the case of PEG-*b*-PLLA/PCL, the melting point of PLLA was approximately 122 °C (Fig. S5a in the ESI†). This can be attributed to the PEG counterpart in the copolymer, whose segments may be trapped in the crystalline regions of PLLA domains and act as a diluent, ultimately depressing its *T*_m_. This is in line with the findings of Han *et al*.^31^ regarding the effect of the PCL block in copolymers with PLLA. The melting point of PCL was not affected by the presence of a PEG block (*T*_m_ = 60 °C)^23^ due to its higher affinity for PLLA,^27^ nor was it affected by the presence of PCL_T_. The glass transition temperature (*T*_g_) of PLLA was observed both in PCL_T_-*b*-PLLA/PCL and PEG-*b*-PLLA/PCL as a small shoulder at approximately 55 °C (Fig. S5a in the ESI†), which corresponded to the PLLA amorphous phase.^32^

The crystalline character of PCL_T_-*b*-PLLA/PCL and PEG-*b*-PLLA/PCL was further analyzed by X-ray diffraction (XRD). The spectra show semicrystalline features, with PLLA peaks at 2*θ* = 16.8° and 19.1° and PCL peaks at 21.4 and 23.8°. The XRD diffractogram of PEG-*b*-PLLA/PCL shows the characteristic peaks of PEG at 19.3 and 23.3°. However, the first peak at 19.3° overlaps with the PLLA peaks (Fig. S7 in the ESI†).^27,29,33^

### PEG-*b*-PLLA/PCL amphiphilic properties

**Fig. 3:**
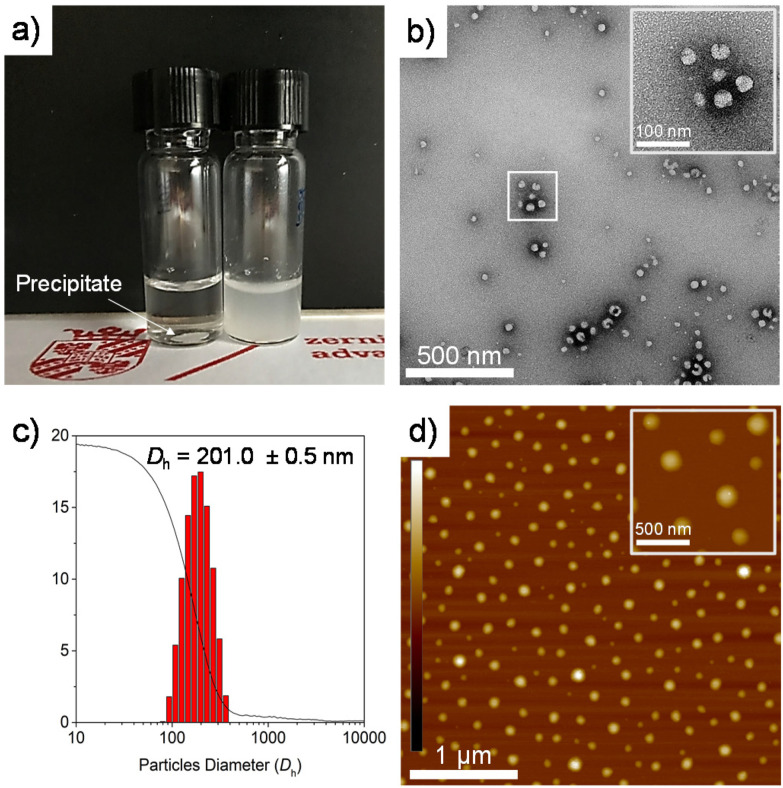
(a) PCL_T_-*b*-PLLA/PCL precipitated (left vial) and PEG-*b*-PLLA/PCL (right vial) in water. (b) TEM images of uranyl acetate-stained self-assembled particles of PEG-*b*-PLLA/PCL and (c) DLS histogram plot and cumulative function (solid line) of PEG-*b*-PLLA/PCL particles. (d) AFM height images of the self-assembled particles deposited on a freshly cleaved mica disk (AFM z-scale is ± 10 nm).

PEG-*b*-PLLA/PCL exhibited amphiphilic properties due to the presence of hydrophilic PEG and more hydrophobic PLLA and PCL in the polymer blend. This amphiphilicity usually results in the self-assembly of polymers into stable structures in aqueous media composed of a hydrophobic core covered by a hydrophilic shell (Fig. 3b).^34^

**Fig. 4:**
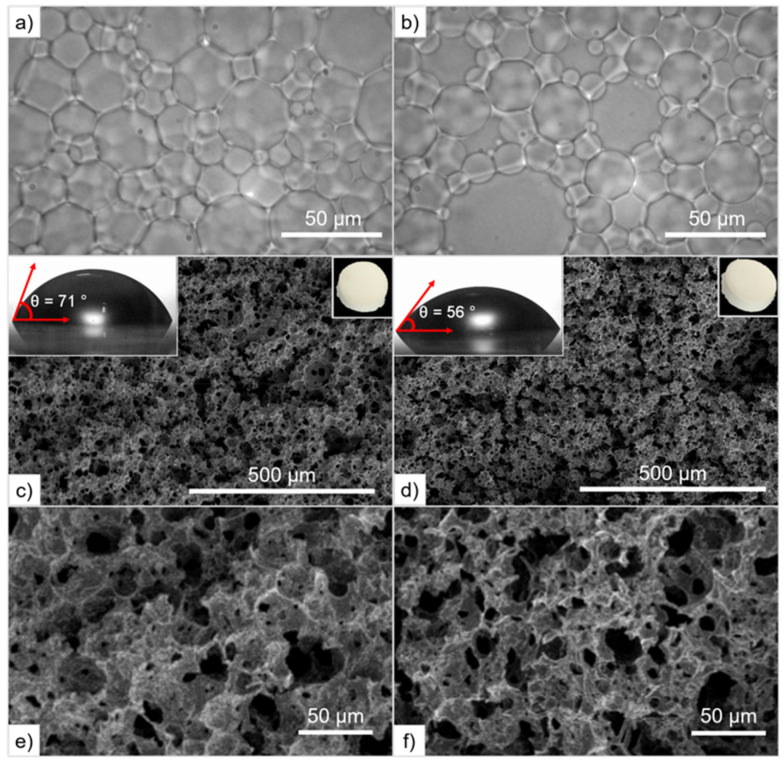
Optical micrographs of oil-in-DESm HIPEs containing different macroinitiators: (a) PCL_T_ and (b) PEG. SEM micrographs of polyHIPEs after extraction of the internal phase: (c) PH(PCL_T_-*b*-PLLA/PCL), and (d) PH(PEG-*b*-PLLA/PCL); inset upper left side shows the water contact angle on polymer films, and inset upper right side shows images of monoliths after purification. SEM micrographs at higher magnification of (e) PH(PCL_T_-*b*-PLLA/PCL), and (f) PH(PEG-*b*-PLLA/PCL).

The morphology of the self-assembled structures resulting from PEG-*b*-PLLA/PCL in aqueous solution was studied by TEM and AFM. Fig. 3b and d confirm the presence of spherical structures of self-assembled PEG-*b*-PLLA/PCL in the range of *ca.* 100 nm. Furthermore, the polyester in aqueous media (15 mg mL^−1^) was studied by dynamic light scattering (DLS), revealing that the spherical structures possessed an average hydrodynamic ratio of 201 nm (Fig. 3c). To measure the hydrophilicity/hydrophobicity properties of PCL_T_-*b*-PLLA/PCL and PEG-*b*-PLLA/PCL, the water contact angle was determined on polymer films prepared by solvent casting. The water contact angle values obtained are listed in Table 1. The contact angle of water of PLLA/PCL blends (BnOH-initiated) is 71°,^19^ while for the PEG-*b*-PLLA/PCL sample, the presence of PEG provides hydrophilic properties, as evidenced by a slightly lower contact angle value of 56° (Fig. 4d). Conversely, the presence of PCL_T_ in PCL_T_-*b*-PLLA/PCL results in a more hydrophobic material because of the inherent properties of PCL, with a contact angle value of 71° (Fig. 4c). These results indicate that macroinitiators can be utilized for tuning the hydrophilic characteristics of polyesters either in bulk or as part of polyHIPEs, as will be discussed in the next sections.

### Synthesis of macroporous polyesters

PLLA and PCL polyesters are hydrophobic materials, which makes them suitable sorbents for the efficient separation of hydrophobic fluids from water, providing a porous and interconnected structure.^35,36^ In this regard, Pérez-García *et al*.^16^ reported the synthesis of degradable and macroporous three-dimensional (3D) polymers by taking advantage of the liquid nature of LLA_30_-CL_70_ DESm to formulate nonaqueous oil-in-DESm HIPEs. The ROP of the continuous phase and subsequent removal of the internal phase (and catalysts)^19^ allows the preparation of degradable and macroporous 3D polymer replicas. Similarly, the synthesis of macroporous nanocomposites by incorporating nonfunctionalized nanohydroxyapatite exposed on their inner surface was reported.^17^

Herein, HIPEs were formulated with the LLA_30_-CL_70_ DESm, containing PEG and PCL_T_ as macroinitiators and Pluronic® F-127 as a surfactant to stabilize the oil-in-DESm emulsion, all composing the continuous phase, 20 vol% of the total emulsion.^16^ The final product after *n*-hexane/ethanol purification and removal of the internal phase (tetradecane) and surfactant appeared as white solid monoliths (top right insets Fig. 4c and d), and were named PH(PCL_T_-*b*-PLLA/PCL) and PH(PEG-*b*-PLLA/PCL), referring to the macroinitiator used in the ROP, PCL_T_ or PEG.

The morphology of the obtained HIPEs consists of polyhedral and polydisperse close-packed droplets (30 ± 2 μm), separated by a characteristic thin film of continuous phase that macroscopically behave as highly viscous emulsions that do not flow upon inversion of the containing vials. Optical microscopy images of the obtained HIPEs are shown in Fig. 4a and b.

The basic insights into the ROP of LLA_30_-CL_70_ DESm using macroinitiators such as hydrophobicity, diffusion, and molecular weight of the final polyesters studied previously in bulk served as the basis for the synthetic conditions translatable to polymerization of HIPEs. Therefore, the sequential ROP of the DESm catalyzed by DBU and MSA was followed for the ROP of the HIPEs, yielding high conversions of 95 and 96% for PH(PCL_T_-*b*-PLLA/PCL) and PH(PEG-*b*-PLLA/PCL), respectively. These figures are similar to the conversions achieved with the same DESm-based HIPEs but initiated with BnOH (94%).^16^

Fig. 4c–f show SEM images of the internal morphology of fractured monoliths after gold sputtering. The macroporous structure of PH(PCL_T_-*b*-PLLA/PCL) and PH(PEG-*b*-PLLA/PCL) consists of an interconnected pore network (33–42 ± 2 μm), similar in size to the droplet diameter of the parent emulsion, in the case of PH(PEG-*b*-PLLA/PCL), as shown in Table S2 in the ESI.†

Herein, the ROP of the HIPEs results in a larger pore size in the case of PH (PCL_T_-*b*-PLLA/PCL) (Table 2). In contrast, PH(PEG-*b*-PLLA/PCL) exhibited better emulsion stability and a lower degree of openness. The presence of a PEG block in the copolymer improved the emulsion stability during polymerization thanks to its amphiphilic and self-assembling properties demonstrated above, which effectively prevented the emulsion from collapsing. Finally, the resulting *δ*_b_ and *V*_T_ values were similar for PH(PCL_T_-*b*-PLLA/PCL) and PH(PEG-*b*-PLLA/PCL), consistent with other porous polyesters reported in the literature.^16,37^

**Table 2:** Morphological properties, molecular weight (*M*_n_), and melting point (*T*_m_) of PH(PCL_T_-*b*-PLLA/PCL) and PH(PEG-*b*-PLLA/PCL)

Sample	*F*_PLLA_ : *F*_PCL_ a	Pore size (μm)	Degree of openness^b^ (%)	*δ*_b_ c (g cm^−3^)	*V*_T_ d (cm^−3^ g)^e^	*M*_n_ e (g mol^−1^)		*M*_n_ f (g mol^−1^)	*Đ*	*T*_m_ g (°C)	
						PLLA	PCL			PLLA	PCL
PH(PCL_T_-*b*-PLLA/PCL)	53 : 47	42 ± 2	14	0.290	3.4	2217	3538	6390	1.61	131	59
PH(PEG-*b*-PLLA/PCL)	51 : 49	33 ± 2	10	0.281	3.6	2059	5250	6457	1.47	133	60

a The mole fraction of monomers (*F*_i_, where i = PLLA or PCL) was obtained by ^1^H NMR using eqn (S1)–(S3) in the ESI.†

b Degree of openness was estimated using eqn (S7)–(S10) in the ESI† proposed by Pulko and Krajnc.^20^

c *δ*_b_, monolith density.

d *V*_T_, total pore volume.

e *M*_n_ was obtained by ^1^H NMR using eqn (S4)–(S6) in the ESI.†

f Combined *M*_n_ of PCL and PLLA obtained by SEC in THF.

g Thermal properties calculated by DSC.

**Fig. 5:**
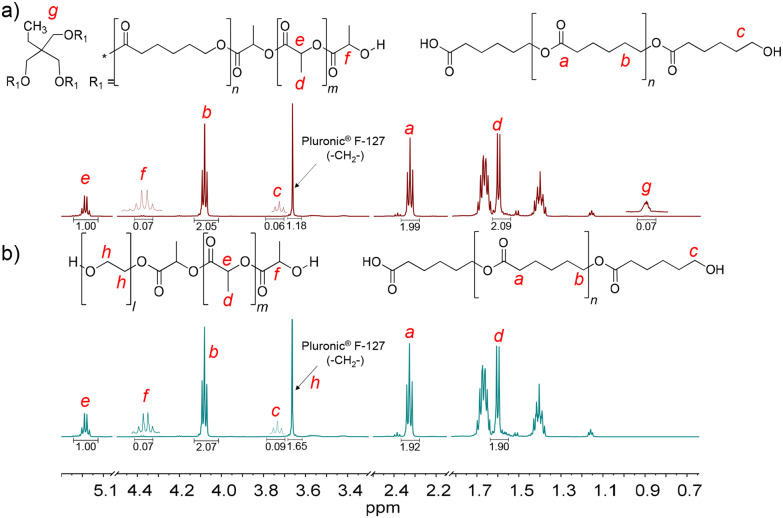
^1^H NMR spectra of polyHIPEs: (a) PH(PCL_T_-*b*-PLLA/PCL) and (b) PH(PEG-*b*-PLLA/PCL).

^1^H NMR spectra of PH(PCL_T_-*b*-PLLA/PCL) and PH(PEG-*b*-PLLA/PCL) are shown in Fig. 5, and similar signals to the polyesters resulting from the ROP in bulk (PCL_T_-*b*-PLLA/PCL and PEG-*b*-PLLA/PCL) were identified. The presence of PLLA blocks in branched PCL_T_-*b*-PLLA or linear PEG-*b*-PLLA copolymers and PCL homopolymer was confirmed by the appearance of the characteristic peaks *H*_e_ and *H*_d_ for PLLA, and *H*_a_ and *H*_b_ for PCL, as discussed above. Interestingly, the surfactant Pluronic® F-127 remained in PH(PCL_T_-*b*-PLLA/PCL) and PH(PEG-*b*-PLLA/PCL), as confirmed by the methylene peak at 3.66 ppm (–C**H**_2−_). Its presence was likely caused by the chains entangled in the branched PLLA and promoted by their affinity for the PEG, which was present in the PEG-*b*-PLLA copolymer. The ^13^C NMR spectra of PH(PCL_T_-*b*-PLLA/PCL) and PH(PEG-*b*-PLLA/PCL) also confirm that the PCL and PLLA ester groups correspond to carbon peaks *C*_1_ = 173 and *C*_8_ = 169 ppm, respectively, and the peak at 70 ppm corresponds to the (–**C**H_2_–) group of the surfactant Pluronic® F-127 (Fig. S11 in the ESI†). The mol fraction (*F*_i_) of PLLA and PCL blocks in PH(PCL_T_-*b*-PLLA/PCL) and PH(PEG-*b*-PLLA/PCL) was calculated by ^1^H NMR spectroscopy (Table 2, eqn (S1)–(S3)†), which also corresponds to the *F*_i_ of polyesters obtained in bulk.

**Fig. 6:**
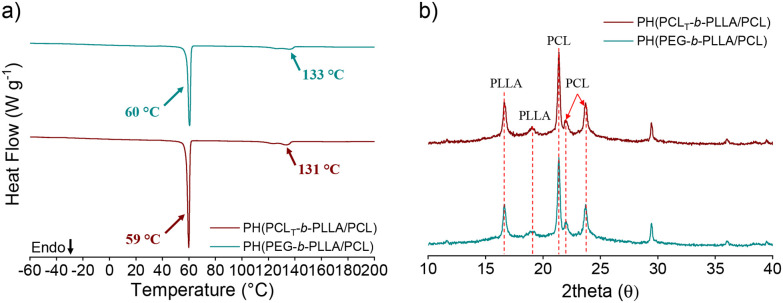
(a) DSC thermograms during the first heating cycle and (b) XRD pattern of PH(PCL_T_-*b*-PLLA/PCL) and PH(PEG-*b*-PLLA/PCL) polyHIPES.

The thermal behaviors of PH(PCL_T_-*b*-PLLA/PCL) and PH(PEG-*b*-PLLA/PCL) were recorded during the DSC heating run (Fig. 6 and Table 2). The *T*_m_ of PLLA is higher in PH(PEG-*b*-PLLA/PCL) than that of PEG-*b*-PLLA/PCL obtained in bulk. The residual surfactant within the polyHIPE contributes to the increment in the melting point due to a favorable interaction with PLLA. In this regard, Athanasoulia *et al.*^35^ observed an improvement in the melting point of PLLA in the presence of PEG. In our case, the increase in the melting point of PH(PEG-*b*-PLLA/PCL) of 133 °C results from the collective effect of PEG in the macroinitiator and the additional surfactant. However, the *T*_m_ of the surfactant (*ca.* 56 °C) in Fig. 6a is not visible, suggesting that the surfactant chains were embedded in the matrix, thus overlapping with the thermal transition of the polyesters. Fig. 6b displays the XRD patterns, where the PEG peaks may overlap with the PLLA and PCL patterns, confirming a possible entanglement with the polyester chains. SEC traces of PH(PCL_T_-*b*-PLLA/PCL) and PH(PEG-*b*-PLLA/PCL) were both observed as broad peaks with an additional peak at low elution times, corresponding to the surfactant Pluronic® F-127 in the samples (Fig. S12 in the ESI†).

### Degradability test

We successfully obtained PH(PCL_T_-*b*-PLLA/PCL) and PH(PEG-*b*-PLLA/PCL) macroporous polyesters, which were primarily composed of a blend of PCL homopolymer with their corresponding branched PCL_T_-*b*-PLLA or linear PEG-*b*-PLLA, respectively.

These materials possess desirable properties such as biodegradability and biocompatibility due to the constituent polymers PCL, PLLA, and PEG blocks, along with the greener ROP utilized. To assess the degradability of PH(PCL_T_-*b*-PLLA/PCL) and PH(PEG-*b*-PLLA/PCL), *in vitro* degradation profile tests were performed. The degradation assay of PH(PCL_T_-*b*-PLLA/PCL) and PH(PEG-*b*-PLLA/PCL) was performed in parallel with the porous polyester composed of PLLA and PCL homopolymer blends initiated by BnOH, namely, PH(PLLA/PCL).

**Fig. 7:**
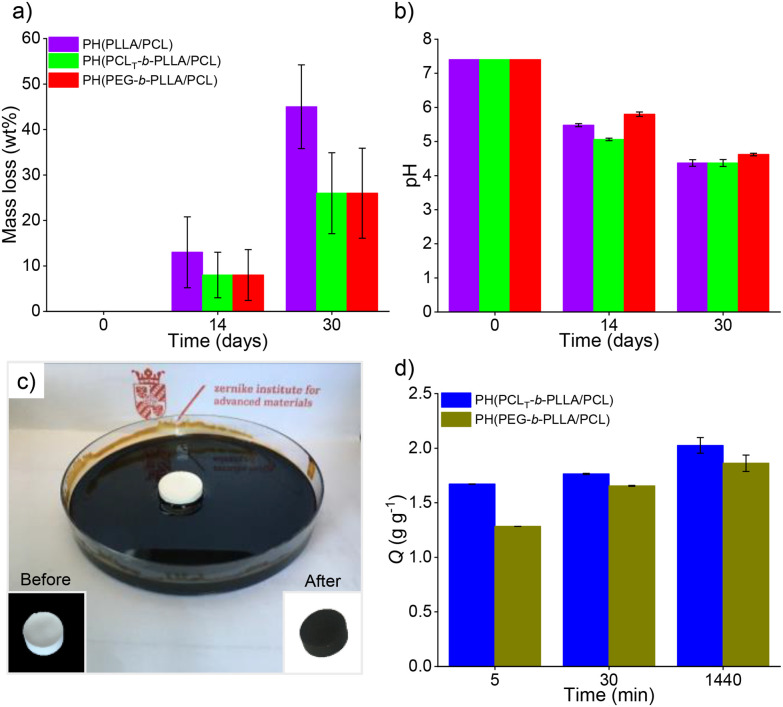
(a) Mass loss (wt%) and (b) pH values of PBS solution profile after degradation of the samples for 14 and 30 days at 37 °C for PH(PCL-PLLA), PH(PCL_T_-*b*-PLLA/PCL) and PH(PEG-*b*-PLLA/PCL) polyHIPES. (Sink conditions, 0.6 wt/v% polyHIPE/PBS solution) (c) Photograph of the crude oil absorption test (inset: monoliths before and after the test, left and right, respectively), and (d) average crude oil absorption of PH(PCL_T_-*b*-PLLA/PCL) and PH(PEG-*b*-PLLA/PCL).

Polyesters degradation in aqueous media can be caused by physical erosion or by cleavage of hydrolytic labile ester bonds. The latter shows an autocatalytic effect due to the hydrolysis of their acidic byproducts, which further accelerates the degradation process. Fig. 7a illustrates the mass loss of the porous polyesters PH(PCL_T_-*b*-PLLA/PCL), PH(PEG-*b*-PLLA/PCL) and PH(PLLA/PCL) after 14 and 30 days of the degradation assay.

The degradation profile showed that the mass loss for all samples at 14 days and 30 days was 10% and 50% for PH(PLLA/PCL), respectively. For PH(PCL_T_-*b*-PLLA/PCL) and PH(PEG-*b*-PLLA/PCL), the mass loss was 30% after 30 days.

Polyester mass loss is primarily due to physical erosion, and factors such as polymer architecture, molecular weight, and hydrophobicity can alter the degradation profile. Therefore, the presence of branched PCL_T_-*b*-PLLA or linear PEG-*b*-PLLA copolymer in PH(PCL_T_-*b*-PLLA/PCL) and PH(PEG-*b*-PLLA/PCL), respectively, reduced erosion by improving the compatibility of the blend constituents.^38^ The mass loss in polyesters was also accompanied by hydrolytic degradation due to acidification of the medium (initial pH of 7.4), causing a decrease in the pH to 4.5, as expected. PH(PCL_T_-*b*-PLLA/PCL) and PH(PEG-*b*-PLLA/PCL) presented lower mass loss than sample PH(PLLA/PCL) after 30 days (Fig. 7b). The presence of branched PCL_T_-*b*-PLLA or linear PEG-*b*-PLLA copolymers in the porous materials provided a better affinity of the polyester's constituents confined into the thin layers of the HIPE polymer replica, therefore exhibiting a lower mass loss compared to PH(PLLA/PCL). In addition, we also observed that the PH(PCL_T_-*b*-PLLA/PCL) and PH(PEG-*b*-PLLA/PCL) monoliths were able to maintain their structures after 30 days of the degradation test, whereas the PH(PLLA/PCL) structure collapsed at the end of the test (Fig. S13 in the ESI†), despite the final acidic pH in all samples.

### Oil adsorption

Macroporous polyesters, such as PH(PLLA/PCL) initiated by BnOH, have been previously reported for removing hydrocarbons from water.^16^ Other porous materials have also been utilized to remove various contaminants from water, such as oil spills.^8^ However, addressing oil spill issues is complex and requires different methods for different scenarios, each with particular cost and time requirements, including environmental impact assessment. Although different materials have been used for crude oil removal, it is necessary to have different alternatives that preferably can be combined to improve their performance.^39,40^ Additionally, the vast majority of sorbents have some drawbacks in terms of lack of degradability; after fulfilling their function, they become another source of waste.^16^ Thus, the development of degradable sorbents is currently needed. PH(PCL_T_-*b*-PLLA/PCL) and PH(PEG-*b*-PLLA/PCL) macroporous polyesters having high porosity and surface area, and synthesized without metal catalysts and at low temperature represent a suitable class of sorbent materials for crude oil. Although some other porous polymers have been reported in the literature, the majority are not degradable,^37^ in contrast to the porous polyesters suggested in this work as a greener alternative.

The crude oil sorption test was performed as shown in Fig. 7c. The capacities of crude oil sorption (*Q*) of PH(PCL_T_-*b*-PLLA/PCL) and PH(PEG-*b*-PLLA/PCL) (*Q*, which represents the mass of crude oil after sorption per gram of dry monolith) were 1.86 ± 0.075 and 2.03 ± 0.071 g g^−1^ after 30 min and 1440 min (Fig. 7d), respectively. The sorption process consists of the accumulation of sorbate on the internal surface and occurs through two phenomena: (i) internal diffusion onto the surface and (ii) retention of the oil molecules by capillarity. The PH(PCL_T_-*b*-PLLA/PCL) sample had a higher degree of openness (Table 2), and the diffusion rate and retention capacity were slightly higher in comparison to PH(PEG-*b*-PLLA/PCL), although both were able to rapidly sorb the crude oil, surpassing 80–70% of their respective *Q* in 5 min.

PH(PCL_T_-*b*-PLLA/PCL) and PH(PEG-*b*-PLLA/PCL) have an absorption capacity for crude oil with a performance comparable to that of other biodegradable materials synthetized by different methods already reported in the literature, such as kapok fibers, lignin, cellulose foam, and cotton. Similarly, when comparing them with nonbiodegradable polyHIPES, such as poly(styrene-codivinylbenzene) polyHIPE, the oil absorption capacity is slightly below those reported by Carranza *et al.*^41^ This could be attributed to the pore openness in the polyHIPES, which regulates the absorption capacity and times, where greater pore openness leads to faster absorption and increased absorption capacity.^40–42^ (Table S3 in the ESI†) As previously discussed, the intrinsic biodegradability and high porosity of these materials make them promising for potential applications in oil spills. They have been observed to degrade with a mass loss of approximately 30% in 30 days, indicating their prospective for full compostability. Last, once they have fulfilled their function of absorbing oil, no additional steps would be necessary for their separation, and their components could become part of the final products.

## Conclusions

In this study, polycaprolactone triol (PCL_T_) and polyethylene glycol (PEG) OH-terminated multifunctional macroinitiators were successfully utilized in the ROP of the LLA_30_-CL_70_ DESm to produce polyesters of diverse macromolecular architectures. The syntheses were performed at 37 °C through a sustainable approach employing organocatalysts, such as DBU and MSA. The resulting polyesters comprised blends of PCL homopolymer with branched or linear PLLA block copolymers, depending on the macroinitiator that selectively initiated the LLA DESm counterpart. These polyesters exhibited different diffusion, hydrophobicity, and molecular weights, which are conducive to controlled degradability.

The incorporation of macroinitiators in the ROP of the LLA_30_-CL_70_ DESm significantly improved the properties of the resulting polyesters, enabling the preparation of macroporous polyesters through emulsion templating. Stable HIPEs oil-in-DESm sustained the efficient organocatalyzed ROP of the polymerizable continuous phase at low temperatures, achieving high conversions and yielding macroporous and interconnected polyester replicas of the emulsions. The obtained macroporous polyesters were degradable while maintaining their macroporous structure for at least 30 days in the *in vitro* degradation test. Additionally, these macroporous materials demonstrated an ability to sorb crude oil, exhibiting competitive sorption capacity figures of 2 g g^−1^.

The bulk polyesters and their corresponding macroporous polyesters obtained through emulsion templating, synthesized using greener ROP under mild temperature conditions, represent alternative materials with promising applications in separation processes, scaffolds for cell culture, and drug delivery.

## Author contributions

Conceptualization: JD M-M, M C–S. Data curation: M C–S, P Q-A, D M. Formal analysis: M C–S, P Q-A., D M, JD M-M, K L. Funding acquisition: JD M-M, K L. Investigation: M C–S, P Q-A., JR T-L, MC G, T P, AJJ W, Q C, MG P-G. Methodology: M C–S, JR T-L, MC G, T P, AJJ W, Q C, MG P-G. Resources: JD M-M, K L. Supervision: D M, JD M-M, K L. Validation M C–S, P Q-A, JR T-L, MC G, T P, AJJ W, Q C, MG P-G. Writing – original draft: M C–S, P Q-A, D M. Writing – review & editing: JD M-M, K L, MC G, P Q-A, MG P-G.

## Conflicts of interest

The authors declare that they have no known competing financial interests or personal relationships that could have appeared to influence the work reported in this paper.

## Supplementary Material

LP-002-D3LP00232B-s001
