# Supplementary material for: Ring-opening polymerization of emulsion-templated deep eutectic system monomer for macroporous polyesters with controlled degradability
Source: RSC Appl Polym. 2024 Feb 5;2(3):403–14. doi: 10.1039/d3lp00232b (PMC11114569; doi:10.1039/d3lp00232b)
Supplement: LP-002-D3LP00232B-s001 [file LP-002-D3LP00232B-s001.pdf]

Electronic Supplementary Material (ESI) for RSC Applied Polymers.
This journal is © The Royal Society of Chemistry 2024

Electronic Supplementary Information

Ring-opening polymerization of emulsion-templated deep eutectic system monomer for macroporous polyesters with controlled degradability

Martín Castillo-Santillan [a,b], Priscila Quiñonez-Angulo [a], Dina Maniar [b], José Román Torres-Lubian [c], María C. Gutiérrez [d], Théophile Pelras [b], Albert J. J. Woortman [b], Qi Chen [b], María Guadalupe Pérez-García [e], Katja Loos [b,*], Josué D. Mota-Morales [a,*]

[a] *Centro de Física Aplicada y Tecnología Avanzada, Universidad Nacional Autónoma de México, Querétaro, QRO 76230, México.*
[b] *Macromolecular Chemistry and New Polymeric Materials, Zernike Institute for Advanced Materials, University of Groningen, 9747AG Groningen, The Netherlands.*
[c] *Centro de Investigación en Química Aplicada (CIQA), Saltillo, Coahuila 25294, México.*
[d] *Instituto de Ciencia de Materiales de Madrid (ICMM), Consejo Superior de Investigaciones Científicas (CSIC), Cantoblanco 28049, Madrid, Spain.*
[e] *Universidad de Guadalajara, Centro Universitario de Tonalá, Tonalá, Jalisco 45425, México.*

**Email: k.u.loos@rug.nl, jmota@fata.unam.mx*

Additional experimental details and photographs of the experimental setup are shown in this file.

SECTION 1. Characterization of the final products of the ROP LLA_{30}-CL_{70} DESm at 37 °C varying PCL_T or PEG as the macroinitiator of polyesters.

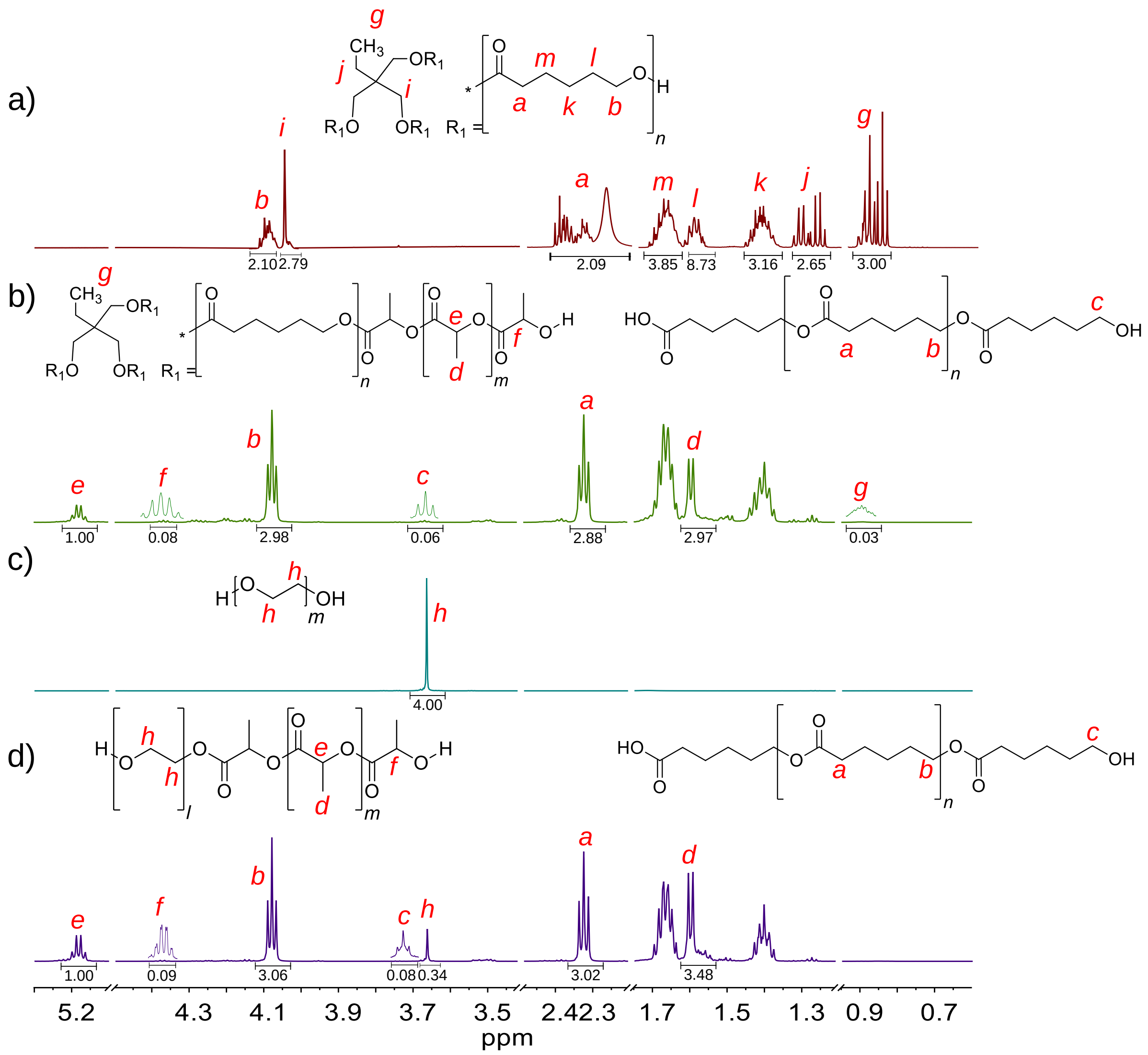

Figure S1. ¹H NMR spectra of the final products of the ROP of LLA_{30}-CL_{70} DESm at 37 °C varying (a) PCL_T or (c) PEG as the macroinitiator of polyesters: (b) PCL_T-*b*-PLLA/PCL, and (d) PEG-*b*-PLLA/PCL.

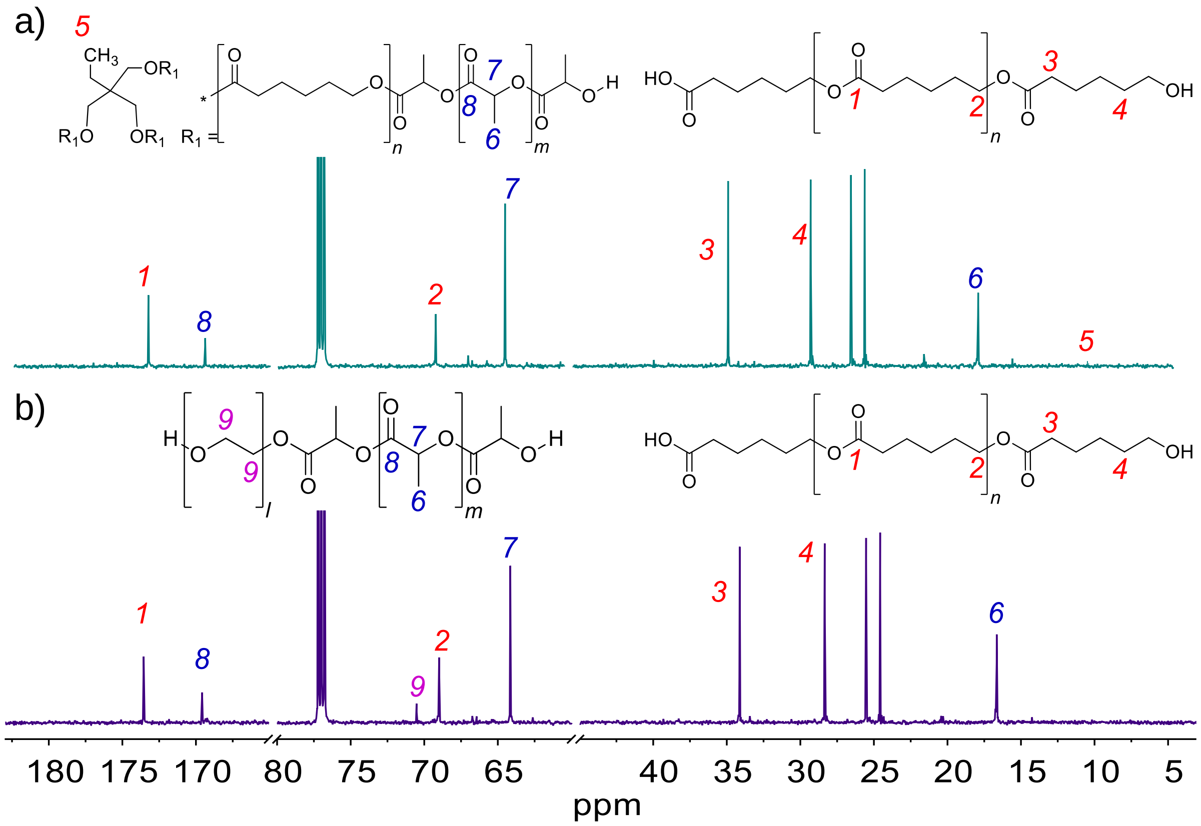

Figure S2. ^{13}C NMR spectra of the final products of the ROP of LLA_{30}-CL_{70} DESm at 37 °C varying PCL_T or PEG as the macroinitiator of polyesters: (a) PCL_T-*b*-PLLA/PCL, and (b) PEG-*b*-PLLA/PCL.

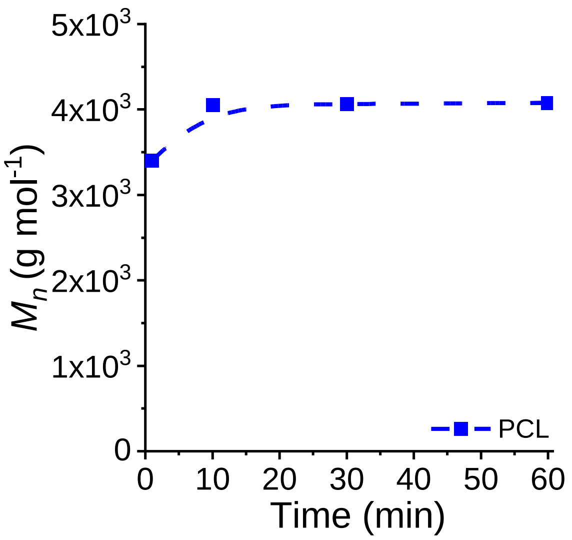

Figure S3. M_n of the PCL produced by adding the MSA organocatalyst at different times after the completion of PLLA ROP (1 min). The M_n was calculated by ^{1}H NMR spectroscopy using the **Eqn. (S6)**.

Equations.

Mole fraction of monomers (F_i, were i = PLLA or PCL) was obtained by ^{1}H NMR.

$$F_{PLLA} = \frac{\int H_e}{\int H_e + \left[\int \frac{H_a}{2} - \left(\frac{\int H_g}{3} * \omega\right)\right]} \tag{S1}$$

$$F_{PLLA} = \frac{\int H_e}{\int H_e + \int \frac{H_a}{2}} \tag{S2}$$

$$F_{PCL} = 1 - F_{PLLA} \tag{S3}$$

Where ω is the number of hydrogen atoms assigned to (-C**H**$_2$-) group (H$_b$) corresponding at the three-arms units of PCL$_T$ with M_n ≈ 900 g mol^{-1}, and in this case, ω = 48. **Eqn. (S1)** was exclusively used when the macroinitiator was PCL$_T$, whereas **Eqn. (S2)** was utilized when PEG was employed.

Molecular weight of polyesters obtained by ^{1}H NMR.

$$M_{n,PLLA}(gmol^{-1}) = \frac{\int H_e}{\int H_f}(MW_{LLA}) \tag{S4}$$

$$M_{n,PCL}(gmol^{-1}) = \frac{\int \frac{H_b}{2} - \left(\int \frac{H_g}{3} * \omega\right)}{\int \frac{H_c}{2}}(MW_{CL}) \tag{S5}$$

$$M_{n,PCL}(gmol^{-1}) = \frac{\int \frac{H_b}{2}}{\int \frac{H_c}{2}}(MW_{CL}) \tag{S6}$$

Where ω is the number of hydrogen atoms assigned to (-C**H**$_2$-) group (H$_b$) corresponding at the three-arms units of PCL$_T$ with M_n ≈ 900 g mol^{-1}, and in this case, ω = 48. **Eqn. (S5)** was exclusively used when the macroinitiator was PCL$_T$, whereas **Eqn. (S6)** was utilized when PEG was employed.

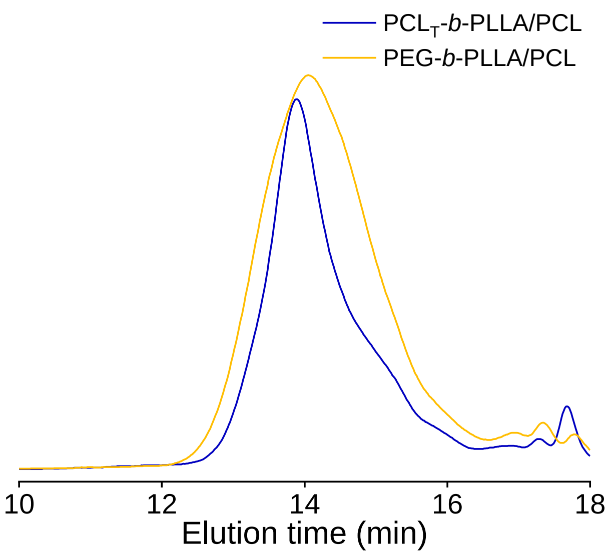

Figure S4. SEC traces of molar mass distribution of branched PCL_T-*b*-PLLA/PCL and linear PEG-*b*-PLLA/PCL polyesters.

The thermal stability of both products, branched PCL_T-*b*-PLLA/PCL and linear PEG-*b*-PLLA/PCL polyesters, was studied by TGA. Branched PCL_T-*b*-PLLA/PCL shows the first degradation at 293 °C with a mass loss of around 7.6%. The second decomposition temperature was observed at 362 °C with a mass loss of 30%. This decomposition is attributed to the degradation of PLLA in the blend, which is consistent with those reported for pure PLLA (361 °C).[1] The thermal degradation of PCL occurs at approximately 400 °C. Conversely, PEG block, in the linear PEG-*b*-PLLA/PCL, was observed at 184 °C, as reported in the literature,[1] followed by the total decomposition of PLLA at 351 °C. The proportion of PLLA in the blend corresponded to *ca.* 30%. Finally, the PCL degradation occurred at *ca.* 408 °C. The degradation temperature of PLLA in the branched PCL_T-*b*-PLLA/PCL increased *ca.* 10 °C compared with linear PLLA in PEG-*b*-PLLA/PCL. This can be attributed to the presence of branched PLLA in the PCL_T-*b*-PLLA/PCL sample allowed for entanglement with the linear PCL homopolymer. This, in turn, increased the thermal stability of the copolymer. (**Figure S5b**).

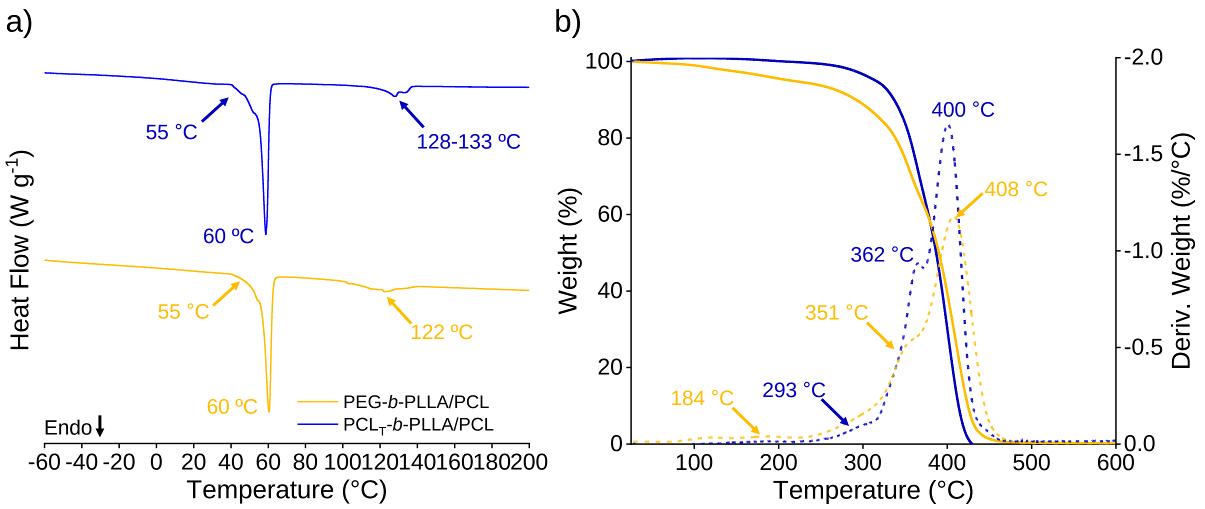

Figure S5. Thermal properties of PCL$_T$-*b*-PLLA/PCL and PEG-*b*-PLLA/PCL polyesters. (a) DSC thermograms during the first heating cycle, (b) TGA (solid lines) and DTGA (dotted lines) curves under nitrogen atmosphere.

ATR-FTIR measurements were conducted on PCL$_T$-*b*-PLLA/PCL and PEG-*b*-PLLA/PCL to confirm the presence of the characteristic functional groups of PLLA and PCL. **Figure S6** shows the peaks at 2943 and 2864 cm^{-1} associated with (-$\mathbf{CH_3}$) and (-$\mathbf{CH_2}$-) groups present in PLLA and PCL.[1] The peak at 1757 cm^{-1} is attributed to (**C=O**) vibration in PLLA.[2] The intensity of this peak decreased in PEG-*b*-PLLA/PCL, suggesting the interaction of PEG counterpart of the block polymers with PLLA. The **C=O** peak attributed to PCL was observed at 1720 cm^{-1}.[3] The peak at 1474 cm^{-1} is attributed to the **C-H** stretching of PEG.[4] The peaks of **-C-O-** and **-COO-** correspond to the presence of PLLA and PCL[3,5] in both PCL$_T$-*b*-PLLA/PCL and PEG-*b*-PLLA/PCL.

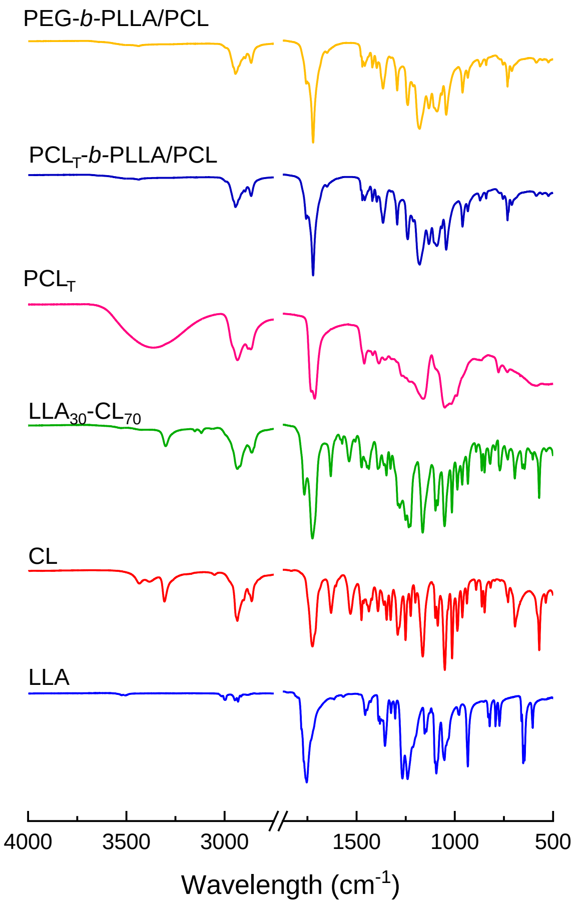

Figure S6. ATR-FTIR spectra of pure monomers LLA and CL, PCL_T, LLA_{30}-CL_{70} DESm and the PCL_T-*b*-PLLA/PCL and PEG-*b*-PLLA/PCL polyesters.

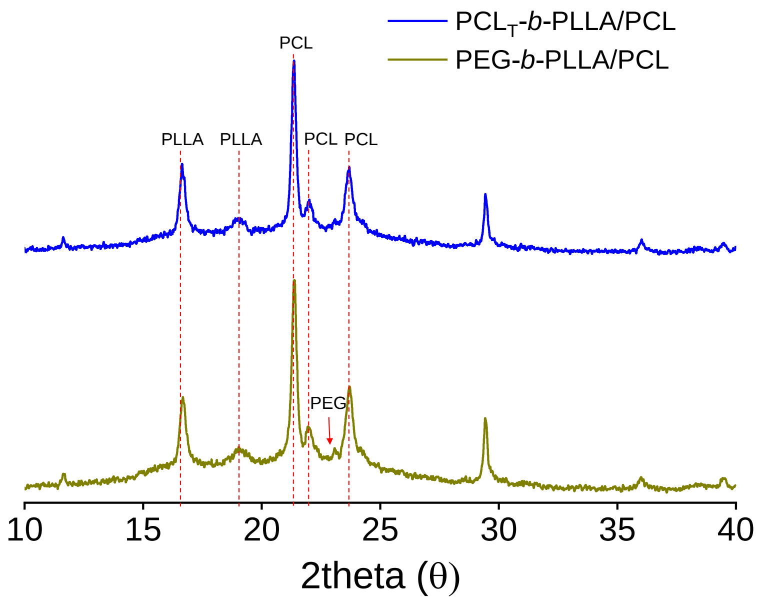

Figure S7. XRD scans of PCL_T-*b*-PLLA/PCL and PEG-*b*-PLLA/PCL polyesters.

SECTION 2. Kinetics study of the ROP LLA_{30}-CL_{70} DESm at 37 °C varying PCL_T or PEG as the macroinitiator of polyesters.

The synthesis of PCL_T-*b*-PLLA/PCL and PEG-*b*-PLLA/PCL polyesters were followed over time, after 6, 8, 12, 24, and 48 h, and the conversion was calculated by gravimetry. **Figure S8** shows the conversion of the PCL_T-*b*-PLLA/PCL sample. Within the first 6 h, the conversion reached around 80%, and full conversion was reached in 24 h. PLLA is formed rapidly in the first stage of the ROP. As the ROP continued, due to the conversion of CL into PCL, the viscosity increased. The reaction temperature (37 °C) was lower than the melting point of PCL in the macroinitiator and the subsequently formed PCL (60 °C); thus, the mobility of the CL monomer was constrained, and the polymerization rate decreased. Conversely, the polymerization of the PEG-*b*-PLLA/PCL sample was slower than PCL_T-*b*-PLLA/PCL; the conversion only reaches 60% after 6 h of reaction and finally reach maximum conversion after 12 h. Altogether, the conversion of both PCL_T-*b*-PLLA/PCL or PEG-*b*-PLLA/PCL reached similar values (*ca.* 80 %) at 12 h. The total polymerization time was longer in comparison to the polyester synthesis reported by Pérez-García *et al.,*[6] which took only 6 h. While a similar DESm and organocatalysts were used, the difference arises from the initiator used in Pérez-García study which is BnOH. The macroinitiators used in this work, PCL_T and PEG, possessed higher molecular weights than BnOH, and the availability of hydroxyl groups, necessary for carrying out the ROP, was constrained. The resulting polyesters in this work possess higher molecular weights than those initiated by BnOH, thus, the viscosity of the reaction mixture was increased and limited the mobility of the monomers during the polymerization.

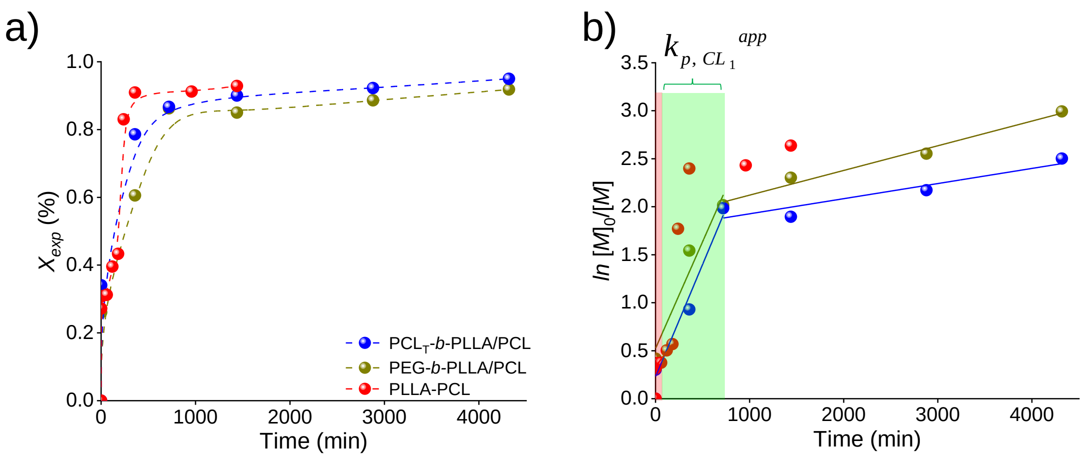

Figure S8. Evolution of the conversion profiles of PCL_T-*b*-PLLA/PCL and PEG-*b*-PLLA/PCL polyesters at 37 °C (Dashed lines denote tendencies, and solid lines are the linear regressions).

Table S1. Estimation of ${k_{p,CL_1}}^{app}$ for LLA_{30}-CL_{70} DESm at 37 °C in bulk varying PCL_T or PEG as the macroinitiator of polyesters.

Sample	Macroinitiator	${k_{p,CL_1}}^{app}$ (s^{-1})	Ref.
PLLA/PCL blend	BnOH	2.23×10^{-5}	[7]
PCL_T-*b*-PLLA/PCL	PCL_T	3.70×10^{-5}	This work
PEG-*b*-PLLA/PCL	PEG	3.90×10^{-5}	This work

To study the role of initiators in PCL_T-*b*-PLLA/PCL and PEG-*b*-PLLA/PCL polymerization, the reaction was followed by ^1H NMR spectroscopy (6, 12, and 24 h). **Figure S9a** shows the ROP of the DESm initiated by PCL_T, where PLLA presence was confirmed by the appearance of the repeating unit peaks at 5.17 and 1.60 ppm, and the methine end group peak at 4.38 ppm. The peak at 0.892 ppm corresponds to the methyl-methylene terminal group of PCL_T. While the peaks at 4.18 and 2.32 ppm are attributed to the PCL repeating methylene group. The terminal methylene group of PCL at 3.66 ppm overlapped with the same terminal methylene groups presented in the PCL_T-*b*-PLLA/PCL, used as macroinitiator. **Figure S9b** shows similar signals corresponding to PLLA and PCL. Additionally, the signal of PEG, that played the role of macroinitiator, at 3.67 ppm overlapped with the PCL end group.

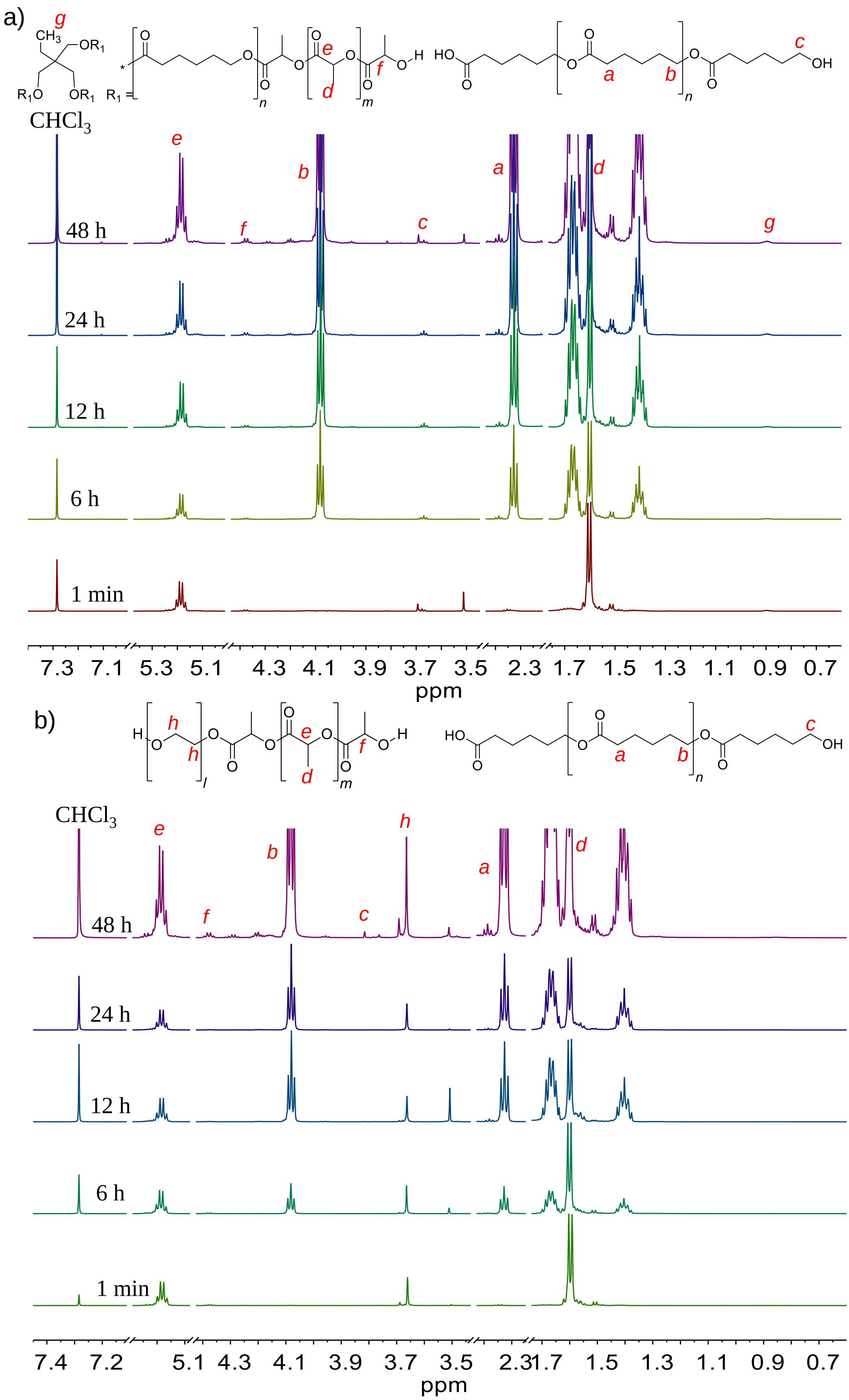

Figure S9. ^{1}H NMR spectra of the sequential ROP of (a) PCL_T-*b*-PLLA/PCL and (b) PEG-*b*-PLLA/PCL.

The presence of branched PLLA or linear PEG copolymers in blends with PLLA was reported to improve their properties, such as flow behavior or tensile strength, respectively.[8,9] We compared the chromatographic profile of branched PCL_T-*b*-PLLA/PCL and linear PEG-*b*-PLLA/PCL with PLLA/PCL homopolymer blends and pure PLLA homopolymer by SEC. PLLA/PCL and PLLA were obtained from the method reported by Pérez García *et al.* initiated by BnOH. [6]

Size exclusion chromatography (SEC) was performed on PLLA, PLLA/PCL, branched PCL_T-*b*-PLLA/PCL and linear PEG-*b*-PLLA/PCL. **Figure S10** shows the unimodal SEC traces of PLLA. PCL and PLLA homopolymer mixtures show a bimodal curve representing PCL and PLLA. The SEC of the branched PCL_T-*b*-PLLA/PCL and linear PEG-*b*-PLLA/PCL show unimodal peaks at a lower retention volume compared to the PLLA/PCL blend. No peak was observed at the same elution time of neat PLLA, which suggested that PLLA obtained in PCL_T-*b*-PLLA/PCL and PEG-*b*-PLLA/PCL have higher molecular weight due to PCL_T and PEG-initiated ROP, and are associated with the other polyesters (including the macroinitiators) as discussed in the main manuscript after the ^{1}H NMR and DOSY results.

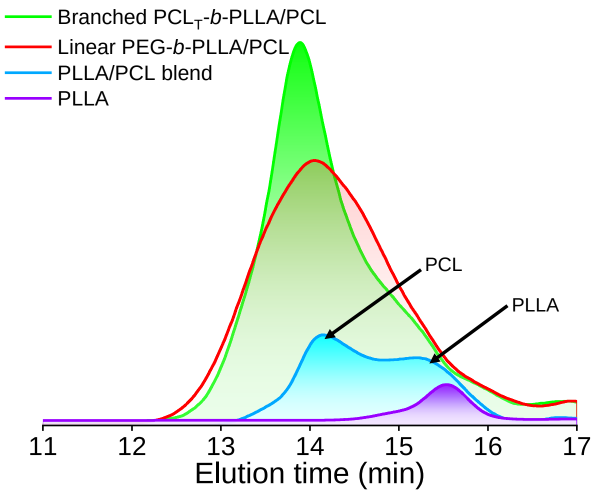

Figure S10. Comparative study of the SEC distribution for PLLA, PLLA/PCL blend, branched PCL_T-*b*-PLLA/PCL and linear PEG-*b*-PLLA/PCL polyesters.

SECTION 3. Characterization of the PH(PCL_T-*b*-PLLA/PCL) and PH(PEG-*b*-PLLA/PCL).

Table S2. Structural morphologies of PH(PCL_T-*b*-PLLA/PCL) and PH(PEG-*b*-PLLA/PCL).

Sample	Droplet diameter (µm)	Pore diameter (µm)	Pore throat (µm)
PH(PCL_T-*b*-PLLA/PCL)	30 ± 2	42 ± 2	12.0
PH(PEG-*b*-PLLA/PCL)	30 ± 2	33 ± 2	7.9

Equations. PolyHIPE openness were estimated using by equation proposed by Pulko and Krajnc.[10]

$$O = \frac{Open\ surface\ of\ cavity}{Surface\ of\ cavity} = \frac{S_O}{S_C} \quad \text{(S7)}$$

$$S_O = N.\pi.\left(\frac{d}{2}\right)^2 \quad \text{(S8)}$$

$$S_C = \pi.D^2 \quad \text{(S9)}$$

$$N = \frac{4\,n}{\sqrt{3}} \quad \text{(S10)}$$

Where:

O = polyHIPES openness

N = number of interconnecting pores

n = average number of visible pore cavities

d = average interconnecting pore diameter

D = average cavity diameter

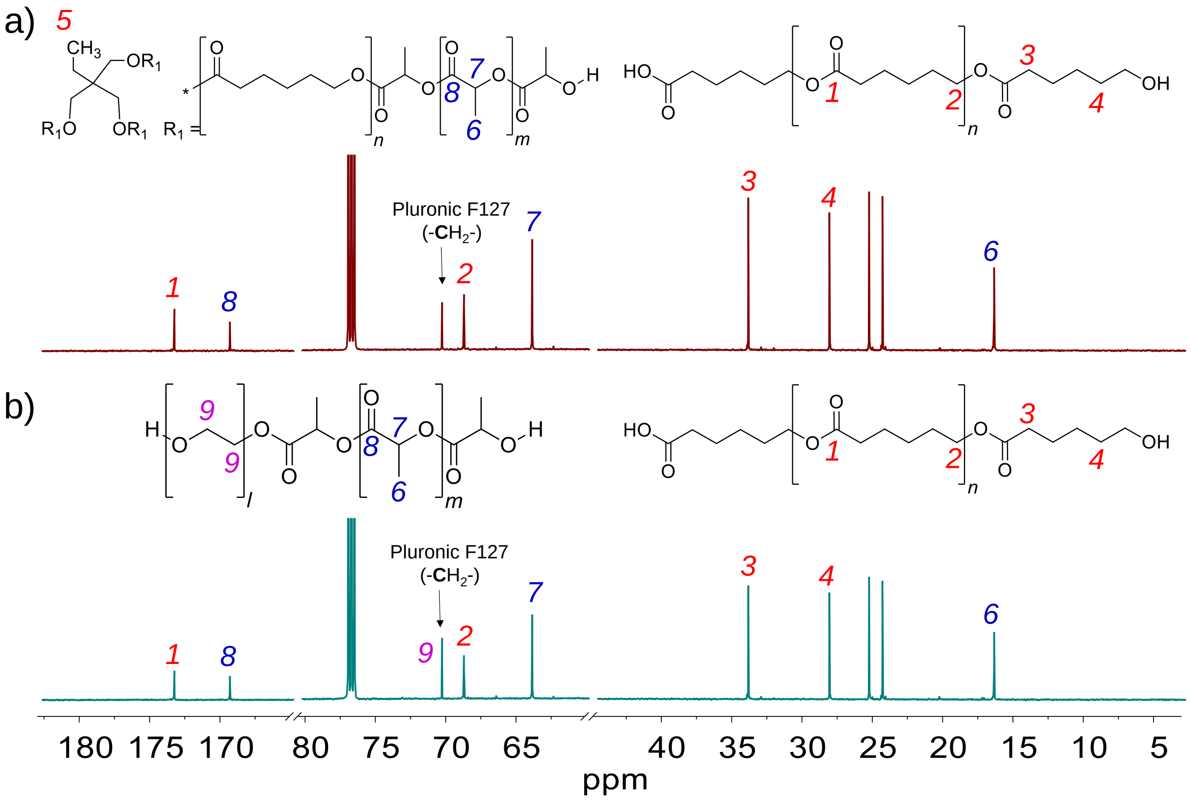

Figure S11. ^{13}C NMR spectra of (a) PH(PCL$_T$-*b*-PLLA/PCL) and (b) PH(PEG-*b*-PLLA/PCL).

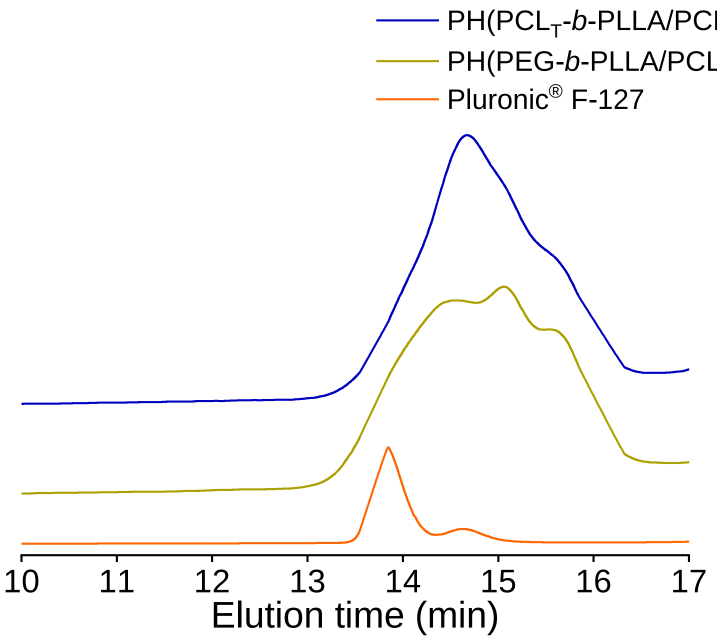

Figure S12. SEC scans of PH(PCL$_T$-*b*-PLLA/PCL), PH(PEG-*b*-PLLA/PCL) and Pluronic® F-127.

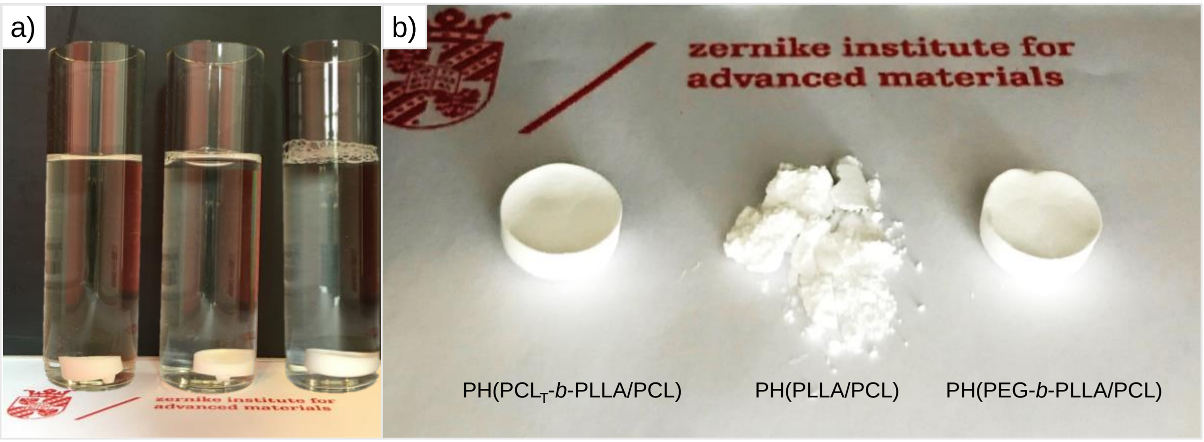

Figure S13. (a) PH(PCL$_T$-*b*-PLLA/PCL), PH(PLLA/PCL) and PH(PEG-*b*-PLLA/PCL) samples, respectively, placed in PBS solution for a day (pH=7.4). (b) Dried samples after 30 days of degradation test.

Table S3. Biodegradable and non-biodegradable materials-based sorbents used for oil adsorption.

Sorbent	Method/Synthesis	Oils	Sorption Capacity (g g^{-1})	Ref.
PH(PCL$_T$-*b*-PLLA/PCL)	polyHIPE	Crude oil	1.86 ± 0.075	This work
PH(PEG-*b*-PLLA/PCL)	polyHIPE	Crude oil	2.03 ± 0.071	This work
Cotton fiber-SiO_2 nanoparticles	Sol–gel process	Hexane<br>Chloroform	25<br>50	11
Poly(butyl methacrylate) and hydrophobic silica on Kapok fibers	Coating fiber	Crude oil	68.3	12
Poly(lactic acid) films	Phase separation process	Petroleum ether	3.5 - 5	13
Lignin-based nanocomposites	Spray casting	Gasoline	2 - 4	14
Cellulose foam	Drop-casting stearic acid and graphite particles	Paraffin<br>Motor oil	9 (v/v)<br>24 (v/v)	15
Poly(styrene-divinylbenzene)	polyHIPE	Biodiesel<br>Diesel<br>Gasoline<br>Hexane	2.71±0.01<br>3.56±0.08<br>4.53±0.09<br>3.36±0.11	16
Vanillin and lauryl methacrylate	polyHIPE	Hexane<br>Toluene<br>Chloroform<br>Kerosene	19.2<br>20.7<br>40.9<br>25.7	17
Silica	polyHIPE	Crude oil	16	18
Sulfonated polystyrene	polyHIPE	Toluene<br>Gasoline	28<br>14	19

References

1 R. Li, Y. Wu, Z. Bai, J. Guo and X. Chen, *RSC Adv.*, 2020, **10**, 42120–42127.

2 X. Yang, S. Liu, E. Yu and Z. Wei, *ACS Omega*, 2020, **5**, 29284–29291.

3 E. M. Abdelrazek, A. M. Hezma, A. El-khodary and A. M. Elzayat, *Egypt. J. Basic Appl. Sci.*, 2016, **3**, 10–15.

4 T. Jayaramudu, G. M. Raghavendra, K. Varaprasad, G. V. S. Reddy, A. B. Reddy, K. Sudhakar and E. R. Sadiku, *J. Appl. Polym. Sci.*, 2016, **133**, 1–8.

5 Viral Tamboli, G. P. Mishra and A. K. Mitra, *Colloid Polym Sci.*, 2013, **291**, 1235–1245.

6 M. G. Pérez-García, M. C. Gutiérrez, J. D. Mota-Morales, G. Luna-Bárcenas and F. Del Monte, *ACS Appl. Mater. Interfaces*, 2016, **8**, 16939–16949.

7 M. Castillo-Santillan, D. Maniar, M. C. Gutiérrez, P. Quiñonez-Angulo, J. R. Torres-Lubian, K. Loos and J. D. Mota-Morales, *ACS Appl. Polym. Mater.*, 2023, **5**, 5110–5121.

8 X. Zhao, J. Li, J. Liu, W. Zhou and S. Peng, *Int. J. Biol. Macromol.*, 2021, **193**, 874–892.

9 I. G. Athanasoulia and P. A. Tarantili, *Pure Appl. Chem.*, 2017, **89**, 141–152.

10 I. Pulko and P. Krajnc, *Macromol. Rapid Commun.*, 2012, **33**, 1731–1746.

11 F. Liu, M. Ma, D. Zang, Z. Gao and C. Wang, *Carbohydr. Polym.*, 2014, **103**, 480–487.

12 J. Wang, Y. Zheng, Y. Kang and A. Wang, *Chem. Eng. J.*, 2013, **223**, 632–637.

13 Z. Xue, Z. Sun, Y. Cao, Y. Chen, L. Tao, K. Li, L. Feng, Q. Fu and Y. Wei, *RSC Adv.*, 2013, **3**, 23432–23437.

14 J. Yang, H. Song, X. Yan, H. Tang and C. Li, *Cellulose*, 2014, **21**, 1851–1857.

15 P. Calcagnile, I. Caputo, D. Cannoletta, S. Bettini, L. Valli and C. Demitri, *Mater. Des.*, 2017, **134**, 374–382.

16 A. Carranza, M. G. Pérez-García, K. Song, G. M. Jeha, Z. Diao, R. Jin, N. Bogdanchikova, A. F. Soltero, M. Terrones, Q. Wu, J. A. Pojman and J. D. Mota-Morales, *ACS Appl. Mater. Interfaces*, 2016, **8**, 31295–31303.

17 H. Zhang, R. Zhao, M. Pan, J. Deng and Y. Wu, *Ind. Eng. Chem. Res.*, 2019, **58**, 5533–5542.

18 D. B. Mahadik, K. Y. Lee, R. V. Ghorpade and H. H. Park, *Sci. Rep.*, 2018, **8**, 1–9.

19 T. Zhang and Q. Guo, *Chem. Eng. J.*, 2017, **307**, 812–819.
